# Activated N-Ras signaling regulates arterial-venous specification in zebrafish

**DOI:** 10.1186/1756-8722-6-34

**Published:** 2013-05-12

**Authors:** Chun-Guang Ren, Lei Wang, Xiao-E Jia, Yi-Jie Liu, Zhi-Wei Dong, Yi Jin, Yi Chen, Min Deng, Yong Zhou, Yi Zhou, Rui-Bao Ren, Wei-Jun Pan, Ting-Xi Liu

**Affiliations:** 1 Key Laboratory of Stem Cell Biology, Institute of Health Sciences, Shanghai Institutes for Biological Sciences, Chinese Academy of Sciences & Shanghai Jiao Tong University School of Medicine, Shanghai, China; 2 Shanghai Institute of Hematology, RuiJin Hospital, Shanghai Jiao Tong University School of Medicine, Shanghai, China; 3 Stem Cell Program, Hematology/Oncology Program at Children’s Hospital Boston, Harvard Medical School, Boston, MA 02114, USA

**Keywords:** Vasculogenesis, Arteriogenesis, N-Ras

## Abstract

**Background:**

The aberrant activation of Ras signaling is associated with human diseases including hematological malignancies and vascular disorders. So far the pathological roles of activated Ras signaling in hematopoiesis and vasculogenesis are largely unknown.

**Methods:**

A conditional Cre/loxP transgenic strategy was used to mediate the specific expression of a constitutively active form of human N-Ras in zebrafish endothelial and hematopoietic cells driven by the zebrafish *lmo2* promoter. The expression of hematopoietic and endothelial marker genes was analyzed both via whole mount *in situ* hybridization (WISH) assay and real-time quantitative PCR (qPCR). The embryonic vascular morphogenesis was characterized both by living imaging and immunofluorescence on the sections with a confocal microscopy, and the number of endothelial cells in the embryos was quantified by flow cytometry. The functional analyses of the blood circulation were carried out by fluorescence microangiography assay and morpholino injection.

**Results:**

In the activated N-Ras transgenic embryos, the primitive hematopoiesis appeared normal, however, the definitive hematopoiesis of these embryos was completely absent. Further analysis of endothelial cell markers confirmed that transcription of arterial marker ephrinB2 was significantly decreased and expression of venous marker flt4 excessively increased, indicating the activated N-Ras signaling promotes the venous development at the expense of arteriogenesis during zebrafish embryogenesis. The activated N-Ras-expressing embryos showed atrophic axial arteries and expansive axial veins, leading to no definitive hematopoietic stem cell formation, the blood circulation failure and subsequently embryonic lethality.

**Conclusions:**

Our studies revealed for the first time that activated N-Ras signaling during the endothelial differentiation in vertebrates can disrupt the balance of arterial-venous specification, thus providing new insights into the pathogenesis of the congenital human vascular disease and tumorigenic angiogenesis.

## Background

RAS proteins are small GTPases that have been proved to be essential for the control of cell proliferation, survival, and differentiation [1,2]. Ras genes are evolutionally and functionally conserved in all eukaryotic organisms. In mammals, the 3 Ras genes encode 4 highly homologous proteins: H-, N-, and K-RAS 4A and 4B. The four RAS proteins interact with a common set of activators and effectors, and thus share many biochemical and biological functions [1]. Both Ras genes and components of Ras signaling pathways, including membrane receptor tyrosine kinases (RTKs), Ras-GEFs, Ras-GAPs, and downstream cytoplasmic kinases (RAFs), are reported to be frequently mutated in diverse human cancers and congenital developmental diseases [3]. In particular, activated mutations of N-Ras gene have been detected in human myeloid leukemias with high frequency [3], ranging from 15% to 60%, and several myeloid leukemia-like mice models were established by inducing the expression of oncogenic N-Ras gene in bone marrow transduction/transplantation [4], transgenic [5] and knock-in [6,7] model systems, suggesting that the oncogenic N-Ras can initiate myeloid leukemias, nevertheless, the long latency and the requirement of cooperative genetic lesions in those models imply that the activated N-Ras signaling alone is insufficient to transform the normal hematopoietic cells into leukemic cells. So far the direct effect of oncogenic N-Ras signaling in hematopoietic cells remains mostly unknown.

Abnormal activation of Ras signaling has been reported to be involved in tumorigenic angiogenesis [8] and congenital vascular developmental diseases [3]. Capillary Malformation-Arteriovenous Malformation (CM-AVM) is a cutaneous congenital vascular disease that is compound of Capillary Malformation (CM) and Arteriovenous Malformation (AVM), and in which the arterial and venous vessels in the skin are connected directly to one another without an intervening capillary bed [9]. Rasa1 gene encodes p120ras-GAP which functions by inhibiting the activity of RAS proteins, and heterozygous inactivating rasa1 mutations were detected and proved to be the causal genetic lesion for CM-AVM [9], suggesting an important role of hyperactive Ras signaling in this disease. So far whether the rasa1 mutations function through an endothelial cell-autonomous manner is still unknown. In addition, the reciprocal signaling between EphrinB2 (artery specific gene) and its receptor EphB4 (vein specific gene) is critical for the formation of capillary beds [10], and it is proposed that a defect in ephrins or their receptors may be a causative factor in the formation of Arteriovenous Malformation (AVM) [11]. And notch pathway mutant (dll4 and Rbpsuh genes) mice embryos exhibit defects in arterial specification of nascent blood vessels and develop Arteriovenous Malformations [12]. However, the pathogenesis of CM-AVM or AVM is largely unknown.

In human N-RAS protein, a point mutation resulting in the substitution of glycine to aspartic acid at codon 12 (G12D) has been proved to impair GAP-stimulated GTP hydrolysis, making the N-Ras signaling constitutively activated [13], hereafter referred as *hNRASD12*. Lmo2 is a transcriptional factor specifically expressed in the hemangioblasts (the bipotential hemato-endothelial progenitor cells) and hemangioblasts-derived primitive hematopoietic and endothelial cells [14,15]. we recently established and functionally characterized the transgenic *Tg(lmo2:Cre)* fish expressing the Cre recombinase under the control of zebrafish *lmo2* promoter [16], and further showed that *Tg(lmo2:Cre)* mediated expression of dominant negative C/ebpα and Bmi1 in zebrafish *lmo2*^*+*^ cells each extended short-lived hematopoietic stem/progenitor cell life span and induced lethal dyserythropoiesis [17]. To explore the roles of aberrant activation of N-Ras signaling in hematopoiesis and vasculogenesis, we used the *Tg(lmo2:Cre)* to mediate the expression of *hNRASD1*2 in *lmo2*^+^ cells of zebrafish embryos, and showed that the activated N-Ras signaling in the *hNRASD1*2 transgenic embryos didn’t induce overt hematopoietic or leukemic phenotypes, while caused severe defective vasculogenesis and subsequently embryonic lethality. The venous cell fate determination was abnormally enhanced at the expense of the arteriogenesis, indicating an endothelial cell-autonomous role of Ras signaling in the arterial-venous specification.

## Results

### Establishment of transgenic zebrafish lines with hemogenic and endothelial cell-specific expression of human oncogenic N-Ras

To address the roles of disease-associated activation of N-Ras signaling in hematopoietic and endothelial systems *in vivo*, we induced specific expression of human *NRASD12* (*hNRASD12*) in these tissues using the Cre-loxP system under the control of *lmo2* promoter in the zebrafish.

**Figure 1:**
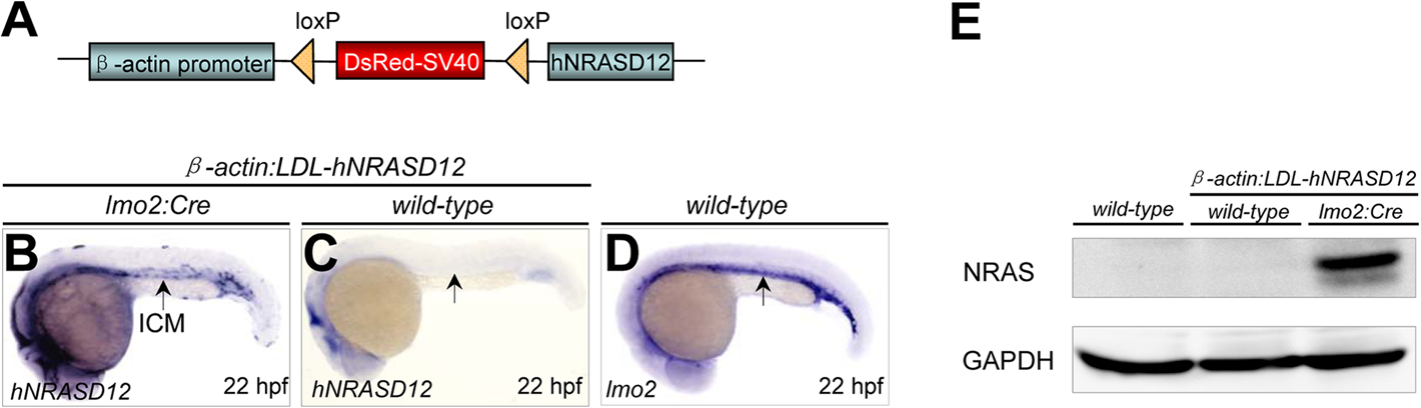
**Validation of *hNRASD12 *expression *in the β-actin:LDL-hNRASD12;lmo2:Cre *transgenic embryos.** (**A**) Diagrammatic scheme of the *β-actin-LDL-hNRASD12* transgenic construct. (**B**-**D**) The expression analyses of the *hNRASD12* transcripts via whole mount *in situ* hybridization (WISH) in the *β-actin:LDL-hNRASD12;lmo2:Cre* embryo (hereafter referred as *hNRASD12* embryo) (**B**) and *β-actin:LDL-hNRASD12;wild-type* embryo (hereafter referred as control embryo) (**C**) respectively at 22 hours post fertilization (hpf), and *lom2* transcripts in the wild-type embryo at 22 hpf (**D**). Black arrows denote the location of the intermediate cell mass (ICM) region. (**E**) Confirmation of the expression of hNRAS protein in the *hNRASD12-*embryos at 28 hpf via western blotting analysis.

We first established the *Tg(β-actin:LDL-hNRASD12)* lines (Figure 1A) and obtained 7 heterozygous stable lines that ubiquitously expressed the fluorescent DsRed protein during embryonic development (data not shown). All 7 lines were crossed to the heterozygous *Tg(lmo2:Cre)* fish individually to generate four different genotypes in the progeny: *hNRASD12*^-^*Cre*^-^ (*wild-type*), *hNRASD12*^-^*Cre*^+^, *hNRASD12*^+^*Cre*^-^ (*β-actin:LDL-hNRASD12;wild-type*) and *hNRASD12*^+^*Cre*^+^ (*β-actin:LDL-hNRASD12; lmo2:Cre).* In particular, the *β-actin:LDL-hNRASD12;lmo2:Cre* double transgenic embryos derived from line 4 showed robust expression of *hNRASD12* transcripts in the intermediate cell mass (ICM) region at 22 hours post fertilization (hpf) (Figure 1B), which mostly resembled the expression pattern of endogenous *lmo2* transcripts in wild-type embryos at the same stage (Figure 1D). And all the *β-actin:LDL-hNRASD12;wild-type* embryos, hereafter referred as control embryos, showed no detectable expression of *hNRASD12* transcripts in the ICM region (Figure 1C). Consistently, the expression of hNRAS protein was only detected in the *β-actin:LDL-hNRASD12;lmo2:Cre* embryos at 28 hpf (Figure 1E), hereafter referred as *hNRASD12* embryos.

### Elevated Ras signaling by overexpression of *hRASD12* has no impact on primitive hematopoiesis

**Figure 2:**
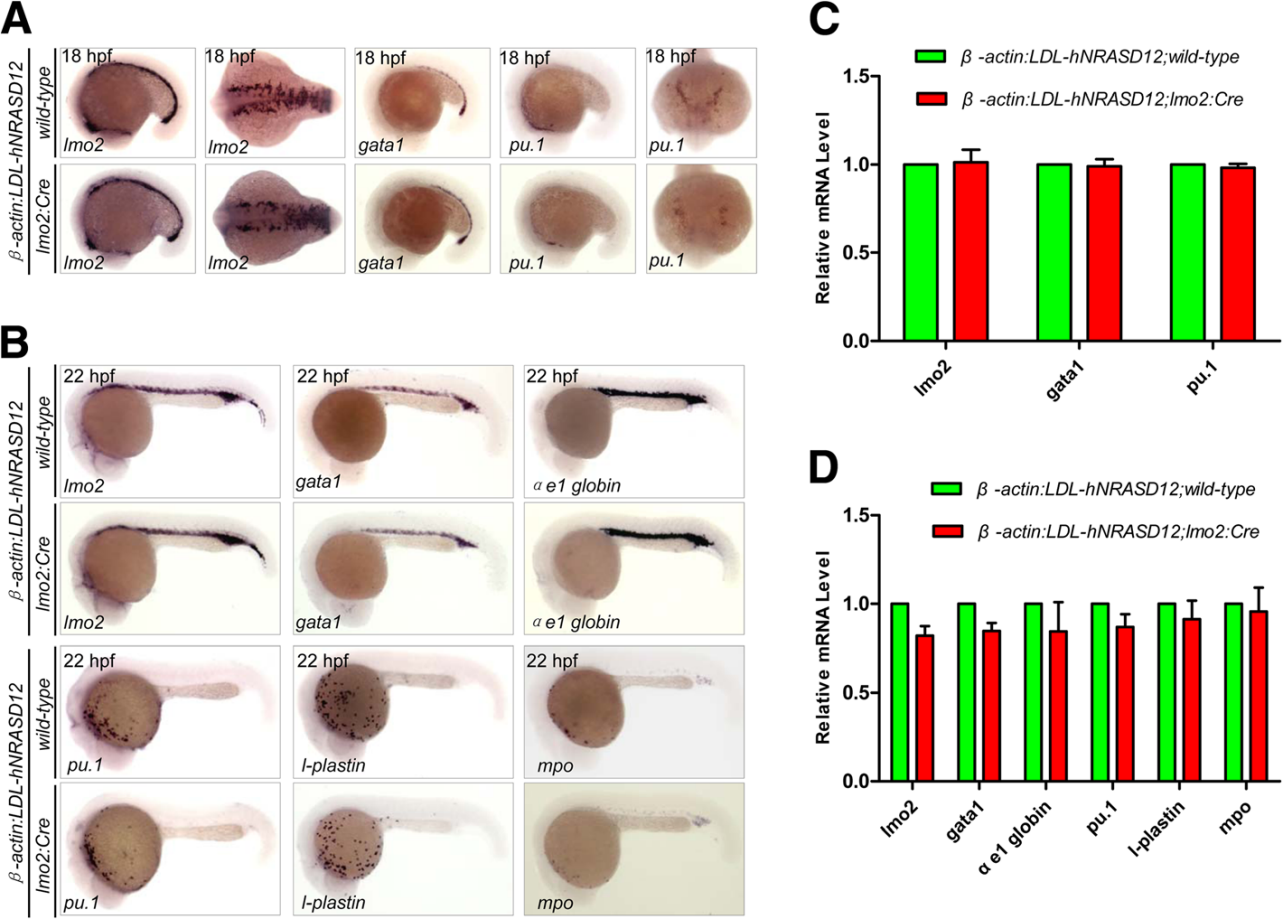
**The primitive erythropoiesis and myelopoiesis are not affected by the expression of *hNRASD12. ***(**A**-**B**) WISH analysis of primitive hematopoietic markers expression in the control embryos and *hNRASD12*expressing embryos respectively at 18 (**A**) and 22 hpf (**B**). (**C**-**D**) Verification of the expression level of the hematopoietic markers in the control or *hNRASD12* embryos at 18 (**C**) and 22 hpf (**D**) via real-time quantitative PCR. Data shown are means ± SEM of at least three independent experiments.

**Figure 3:**
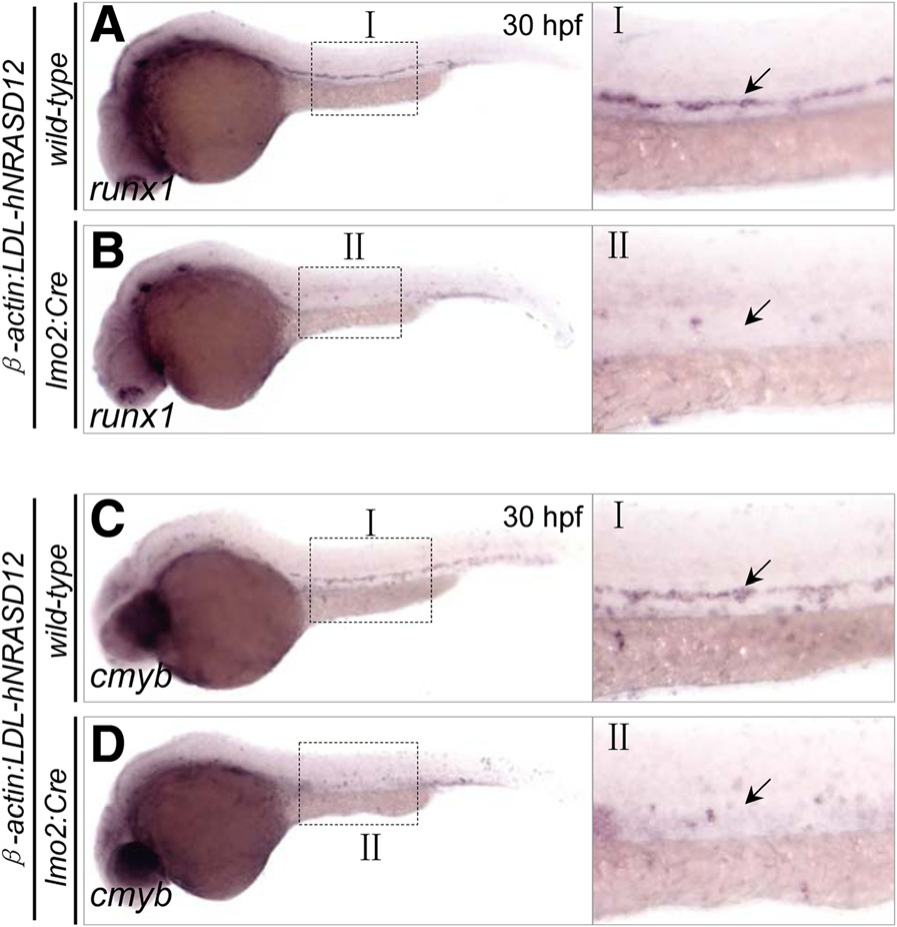
**The definitive hematopoiesis is disrupted in the *hNRASD12*-expressing embryos.** (**A**-**D**) WISH analysis of *runx1*^*+*^ and *cmyb*^*+*^ definitive hematopoietic stem cells (HSCs) in the aorta-gonad-mesonephros (AGM) region of the trunk in the control (**A**, **C**) and *hNRASD12* embryos (**B**, **D**) at 30 hpf (arrows).

After confirmation of the *hNRASD12* expression in the transgenic embryos, we examined the effect of activated N-Ras signaling on the specification and differentiation of blood cells in the *hNRASD12* embryos. Like mammalian, zebrafish hematopoiesis includes two consecutive waves of hematopoiesis: primitive and definitive hematopoiesis [18]. Primitive hematopoiesis originates from the anterior and posterior lateral plate mesoderm and produces erythrocytes, macrophages, and granulocytes, providing blood cells for early embryogenesis [18]. The definitive hematopoietic stem cells (HSCs) emerge from the hemogenic endothelium at the ventral wall of the dorsal aorta from 30 hpf onward, called endothelial hematopoietic transition (EHT) [19,20], and generate all types of blood cells in adulthood [18]. To characterize the hematopoietic phenotypes in the *hNRASD12* embryos, we performed whole mount *in situ* hybridization (WISH) analysis to examine the hematopoietic markers expression [21,22] in the control and *hNRASD12* embryos at 18 and 22 hpf, including *lmo2* (hemangioblasts), *gata1* (primitive erythroid progenitors), *αe1 globin* (mature erythrocytes), *pu.1* (primitive myeloid progenitors), *l-plastin* (primitive macrophages), *mpo* (neutrophils), *cmyb* and *runx1* (HSCs). The results showed that expression of those primitive hematopoietic markers is not affected in the *hNRASD12*-expressing embryos (Figure 2A & B). Consistent with the WISH results, the real-time quantitative PCR (qPCR) analysis showed no significant difference at the expression level of these markers between the *hNRASD12* and control embryos, indicating the activated N-Ras doesn’t alter the development of primitive blood cells (Figure 2C & D). However, the definitive HSCs marked by *runx1* and *cmyb* couldn’t be detected in the aorta-gonad-mesonephros (AGM) region of the *hNRASD12* embryos (Figure 3A-D). According to the defective blood circulation and dorsal aorta formation in the *hNRASD12* embryos described below, the lack of definitive hematopoiesis is likely the secondary effect of the vascular defects, as both the normal blood circulation and dorsal aorta formation have been proven to be indispensable for the emergency of definitive HSCs in the AGM region [23,24], thus preventing from directly dissecting the role of activated N-Ras signaling in definitive hematopoiesis. Taken together, these data show that the oncogenic N-Ras signaling in the zebrafish *lmo2*^+^ cells doesn’t induce directly hematopoietic or leukemic phenotypes in the transgenic embryos.

### Vascular morphogenesis defects in *hNRASD12*-expressing transgenic embryos

**Figure 4:**
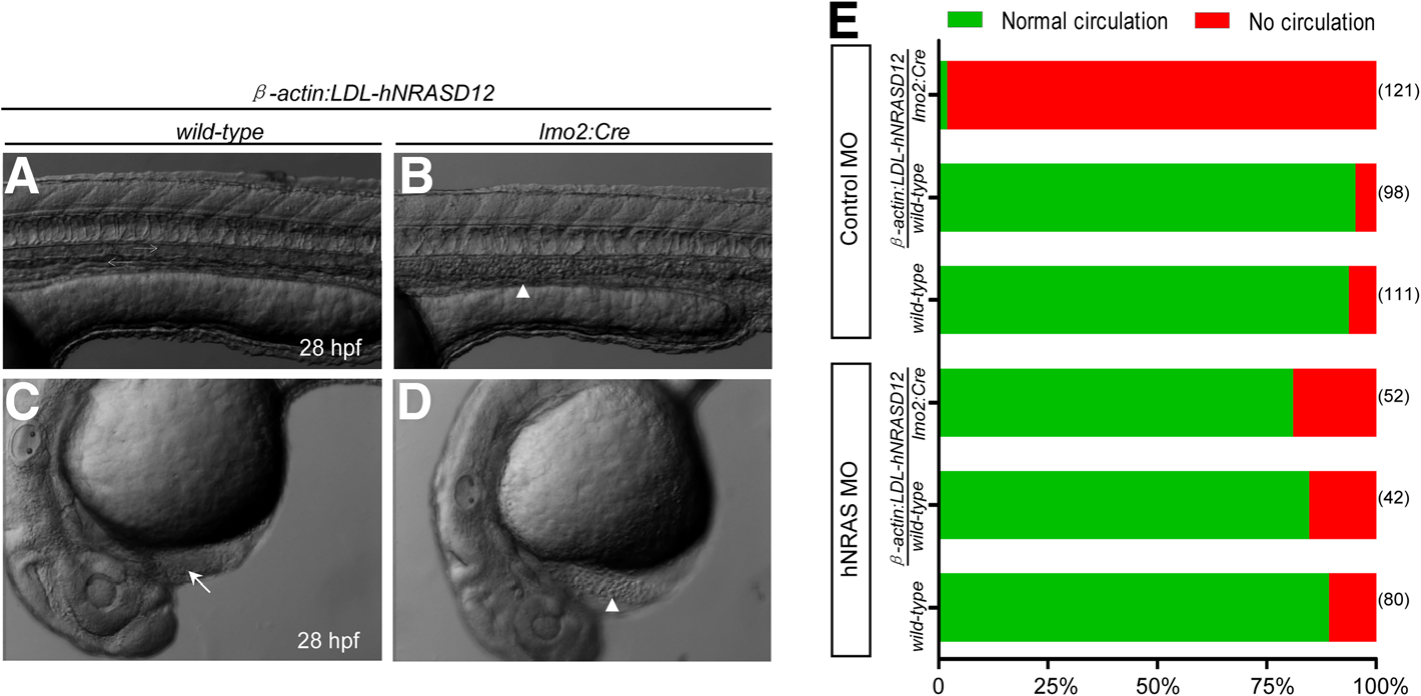
**The expression of *hNRASD12 *blocks the blood circulation.** (**A**-**D**) Morphology of the control (**A** &**C**) and *hNRASD12* embryos (**B** &**D**) at 28 hpf. The arrows mark the blood flow in the trunk and heart of control embryo, and the triangles denote the accumulated blood cells in the trunk and heart of *hNRASD12* embryo. (**E**) The assessment of blood circulation in wild-type, control and *hNRASD12* embryos after injection of control and hNRAS morpholino (MO) respectively; the numbers on the right of the bars indicate the amount of the embryos being counted, and representative results obtained from three independent experiments.

Expression of *hNRASD12* under the *lmo2* promoter will lead to expression both in the hematopoietic and endothelial lineages, so we then examined the circulatory system and found the *hNRASD12* embryos at 28 hpf lacked blood circulation both in head and trunk vessels, and the blood cells were accumulated in the trunk axial vessels and heart chambers (Figure 4B & D, and Additional files 1 and 2: movie S3 and S4) in comparison of normal circulation in the control embryos (Figure 4A & C, and Additional files 3 and 4: movie S1 and S2). The *hNRASD12* embryos died between 5 and 8 days post fertilization (dpf) with the persistent lack of blood circulation. To validate the blockage of circulation was specifically induced by the expression of human *NRASD12*, we designed a rescue experiment by using an antisense ATG morpholino (MO) targeting the translational initiation site of this transgenic *hNRASD12*. Firstly, the efficiency of the hNRAS MO was confirmed by efficiently blocking both the translation of the GFP mRNA reporter (Additional file 5: Figure S1A-D) in wild-type embryos and the expression of hNRASD12 protein in the *hNRASD12* embryos (Additional file 5: Figure S1E). Compared with the control MO, the hNRAS MO that was injected into one-cell stage embryos derived from the crossing between the heterozygous *Tg(lmo2:Cre)* and *Tg(β-actin:LDL-hNRASD12)* fish restored the blood circulation of *hNRASD12* embryos to a comparable level as that of control embryos (Figure 4E), indicating the blocked blood circulation is specifically induced by *hNRASD12* ectopic expression. Considering the primitive hematopoiesis of *hNRASD12* embryos is normal, we hypothesized that the congestion of circulation resulted from the defective cardiovascular development. We then checked the cardiovascular structure and function by using the fluorescent microangiography assay. The fluorescein-coupled latex beads were injected into the inflow tract of the atrium of the control and *hNRASD12* embryos respectively, and were observed to perfuse the whole vascular system of the control embryos in five minutes (Additional file 5: Figure S2A, upper panel). However, it took over 30 minutes for injected beads to passively diffuse into the anterior trunk of the *hNRASD12* embryos (Additional file 5: Figure S2A, lower panel), indicating the activated Ras signaling disrupts the circulation. In particular, the consecutive accumulation of fluorescent beads in the trunk of *hNRASD12* embryos (Additional file 5: Figure S2A, lower panel) suggested the existence of only one axial vascular tube. We further examined the function of the heart of *hNRASD12* embryo by counting the rate of heartbeats, and the result showed that the average rate of heartbeats in the *hNRASD12* embryos at 30 hpf was only slightly slower than that of control embryos (Additional file 5: Figure S2B), suggesting the phenotype of heartbeat contributes little to the circulation blockage.

**Figure 5:**
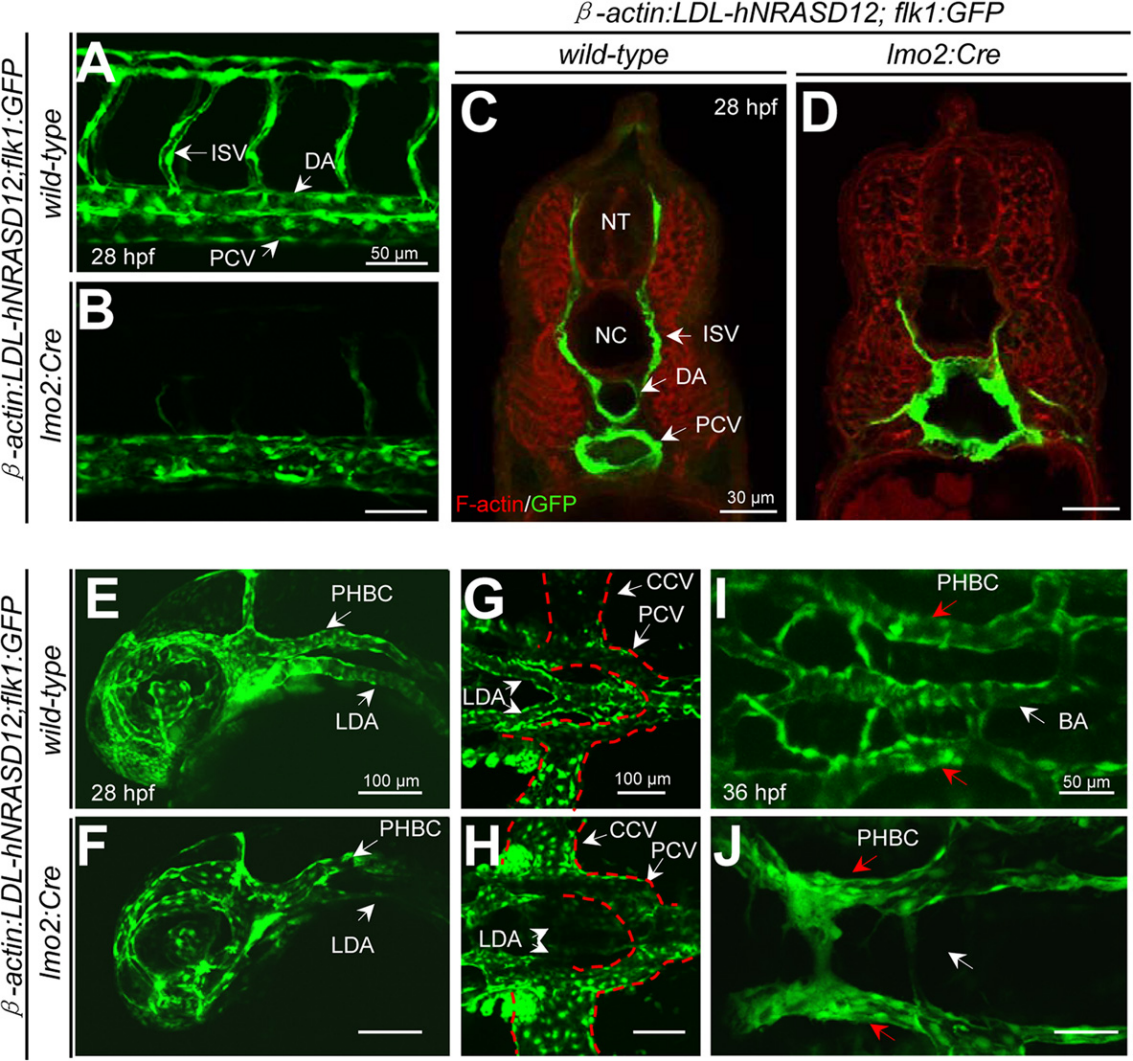
**Vasculogenesis is disrupted in *hNRASD12*-expressing transgenic embryos.** (**A**-**B**) Confocal images of trunk vasculature of living *control; flk1:GFP* (A) and *hNRASD12;flk1:GFP* embryos (**B**) at 28 hpf. DA, dorsal aorta; PCV, posterior cardinal vein; ISV, intersegmental vessel. (**C**-**D**) Mid-trunk transverse sections of *control;flk1:GFP* (C) and *hNRASD12;flk1:GFP* embryos (**D**) at 28 hpf stained by GFP antibody (green) and Phalloidin-TRITC (red). NT, neural tube; NC, notochord. (**E**-**J**) Confocal images of head vasculature of living *control;flk1:GFP* (**E**, **G**, **I**) and *hNRASD12;flk1:GFP* embryos at 28 hpf (**F**, **H**, **J**). LDA, lateral dorsal aorta; PHBC, primordial hindbrain channel; CCV, common cardinal vein; BA, basilar artery. The red dotted lines denote the boundaries of the CCVs and PCVs respectively.

To further analyze the vascular defect in the *hNRASD12* embryos, we crossed the *Tg(flk1:GFP)* fish [25], an endothelial reporter transgenic line, with *Tg(β-actin:LDL-hNRASD12)* fish to obtain the double transgenic progeny, which were raised to adulthood and subsequently crossed to *Tg(lmo2:Cre)* fish to generate the *β-actin:LDL-hNRASD12;flk1:GFP;lmo2:Cre* (referred as *hNRASD12;flk1:GFP*) embryos. In the trunk of *β-actin:LDL-hNRASD12;flk1:GFP;wild-type* (referred as *control;flk1:GFP*) embryos at 28 hpf, the primary blood vessels consisted of the dorsal aorta (DA) and posterior cardinal vein (PCV), and the intersegmental vessels (ISVs) formed by sprouting from the dorsal aorta (Figure 5A) [26]. However, *hNRASD12;flk1:GFP* embryos failed to form morphologically distinct DA or PCV, and the ISVs were also defective (Figure 5B). The results of immunofluorescence analysis on transverse sections showed the presence of only one vascular tube in the trunk of *hNRASD12*-expressing embryo (Figure 5C & D). Furthermore, the morphology of head vasculature was also examined, in the *control;flk1:GFP* embryos at 28 hpf, the paired bilateral lateral dorsal aortas (LDAs) merged backward into single dorsal aorta at the boundary between head and trunk, and the posterior cardinal vein (PCV) split forward into a pair of vessels in the cranial trunk, then the branches of PCV emptied into the common cardinal veins (CCVs) on the either side of yolk, which came into the sinus venous of the heart (Figure 5E & G) [26]. Unlike that of *control;flk1:GFP* embryos, the LDAs of *hNRASD12;flk1:GFP* embryos were evidently atrophic while CCVs and the bifurcation of PCV were greatly expanded, however, the primordial hindbrain channels (PHBCs), the primary veins in the head at this stage, seemed normal (Figure 5F & H). In addition, basilar artery (BA), the main artery in the hindbrain of the zebrafish embryo formed by endothelial sprouting of PHBCs [27], was lost in the *hNRASD12*-expressing embryo (Figure 5I & J). Taken together, our observations suggested that the activated N-Ras signaling in the zebrafish *lmo2*^+^ cells caused the abnormal assembly of the axial blood vessels and the defective arteries formation and expansion of veins in the head vasculature system of the *hNRASD12* embryos.

### *hNRASD12* promotes venous fate specification at the expense of arterial fate in zebrafish

**Figure 6:**
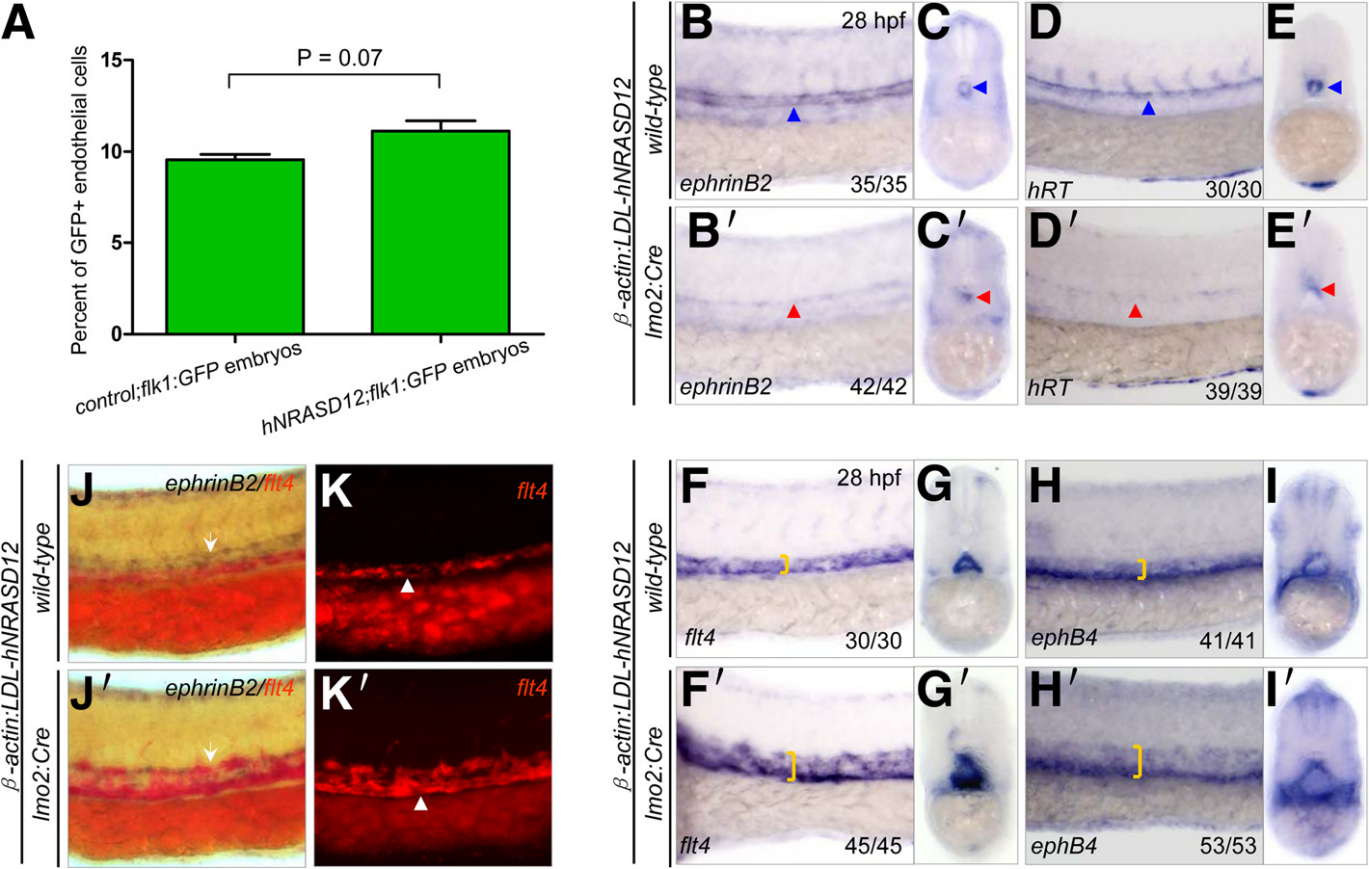
**Expression of *hNRASD12 *promotes venous specification at the expense of arterial fate during embryonic endothelial differentiation.** (**A**) Quantitation of total endothelial cells by flow cytometry analysis on the *control;flk1:GFP* and *hNRASD12;flk1:GFP* embryos at 28 hpf. Representative results obtained from three independent experiments. 2 tailed *t*-test of frequency of endothelial cells in *hNRASD12;flk1:GFP* embryos vs *control;flk1:GFP* embryos shows no significant difference. (**B**-**E**’) WISH analysis of *ephrinB2*^*+*^ and *hRT*^*+*^ arterial cells in the trunk of control (**B**-**E**, blue triangles) and *hNRASD12* embryos (**B**’-**E**’, red triangles) at 28 hpf. (**F**-**I**’) WISH analysis of *flt4*^*+*^ and *ephB4*^*+*^ venous cells in the trunk of control (**F**-**I**) and *hNRASD12* embryos (**F**’-**I**’) at 28 hpf. The yellow square brackets mark the width of the venous strip in the trunk. (**J**-**K**’) 2-color WISH analysis with digoxigenin-labeled *ephrinB2* (white arrows) and fluorescein-labeled *flt4* (white triangles) in control (**J**, **K**) and *hNRASD12* embryos (**J**’, **K**’) respectively at 28 hpf.

As Ras signaling has been proved to be important for cell proliferation [1,2], the frequencies of endothelial cells in the *hNRASD12;flk1:GFP* and *control;flk1:GFP* embryos at 28 hpf were respectively quantified by flow cytometry analysis, and the result showed no significant difference in the frequencies of GFP^+^ endothelial cells between *hNRASD12*-expressing and control embryos (Figure 6A). We further characterized the arterial and venous cells lineages respectively in the *hNRASD12* embryos by performing WISH analysis. *EphrinB2*, *hRT*, *dll4* and *grl* genes are specifically expressed in the arterial cells of wild-type embryo’s trunk region [23,27], while *ephB4*, *flt4* and *dab2* mark the venous cells [28]. The result of WISH showed that the arterial cells were evidently reduced in *hNRASD12*-expressing embryos compared with control embryos (Figure 6B, B’, D, and D’), and failed to form an intact dorsal aorta (Figure 6C, C’, E, and E’); On the contrary, the venous cells in the *hNRASD12* embryos were greatly expanded and organized to be an enlarged vein-like vascular tube (Figure 6F-I and F’-I’). To confirm that expression of *hNRASD12* induced an unbalanced arterial-venous specification in the same embryo, we performed 2-color WISH analysis with digoxigenin-labeled *ephrinB2* and fluorescein-labeled *flt4* antisense probes, and observed the expansion of venous cells and reduction of arterial cells occurring in a single *hNRASD12*-expressing embryo (Figure 6J’ & K’) compared to that in control embryo (Figure 6J & K). These results from WISH with *dll4*, *grl*, and *dab2* probes also supported the above observations (Additional file 5: Figure S3 A-F) that almost the entire vascular tube in the trunk of *hNRASD12* embryo consists of venous endothelial cells.

**Figure 7:**
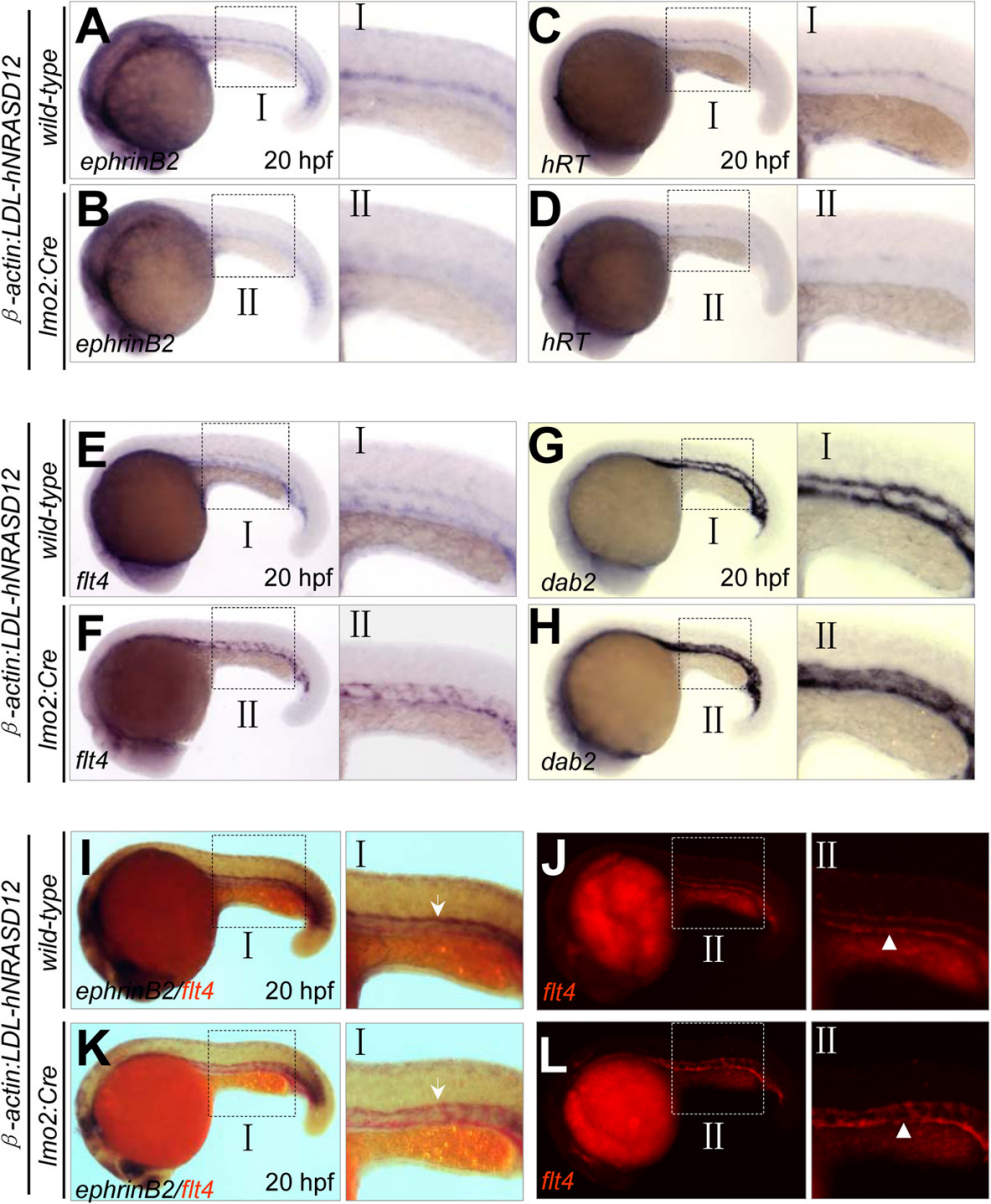
**Loss of arterial specification by *hNRASD12 *expression before arterial-venous segregation and onset of circulation.** (**A**-**H**) WISH analysis of arterial (*ephrinB2*^*+*^, *hRT*^*+*^) and venous (*flt4*^*+*^, *dab2*^*+*^) cells in the trunk of control (**A**, **C**, **E**, **G**) and *hNRASD12* embryos (**B**, **D**, **F**, **H**) at 20 hpf respectively. (**I**-**L**) 2-color WISH analysis with digoxigenin-labeled *ephrinB2* (white arrows) and fluorescein-labeled *flt4* (white triangles) in control (**I**, **J**) and *hNRASD12* embryos (**K**, **L**).

**Figure 8:**
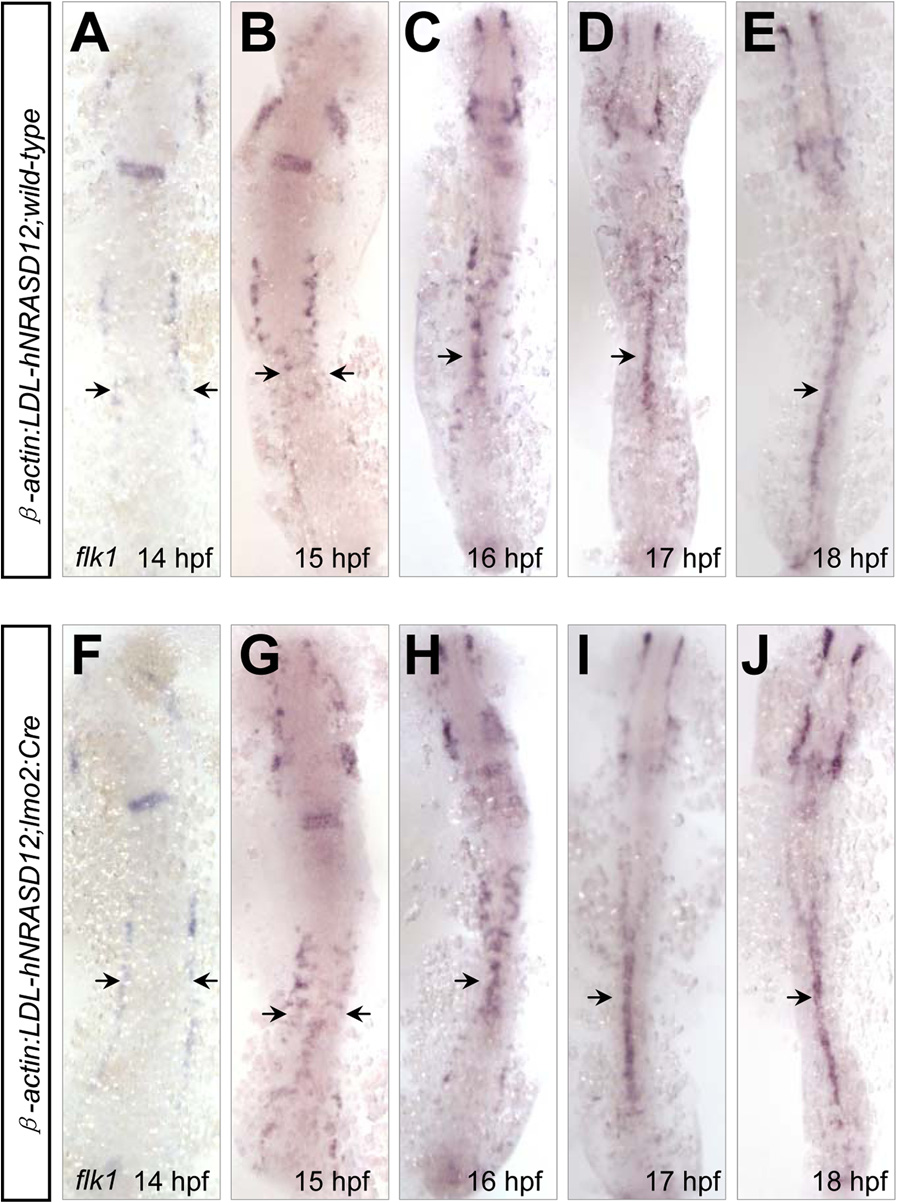
**The medial migration of angioblasts in the posterior bilateral mesoderm is not affected by the activated N-Ras signaling.** (**A**-**J**) The distribution of *flk1*^*+*^ angioblasts at the posterior lateral plate mesoderm in the control (**A**-**E**) and *hNRASD12* embryos (**F**-**J**) from 14 to 18 hpf.

Vasculogenesis is referred to as the de novo formation of the blood vessels, involving the differentiation, proliferation and migration of endothelial cells [29]. In zebrafish, the angioblasts at the bilateral posterior lateral plate mesoderm migrate medially to form the vascular cord between 14 and 18 hpf, meanwhile the arterial-venous specification is established [23,25]. Then the venous cells sprout ventrally to separate from the arterial cells between 21 and 24 hpf, called arterial-venous segregation [30]. Subsequently, the dorsal arterial cells organize to be dorsal aorta by cord hollowing [31], while the posterior cardinal vein forms by embrace of the hematopoietic cells [30]. To figure out which of the above developmental processes is affected firstly by the expression of *hNRASD12*, we used WISH analysis to detect the arterial-venous specification at earlier stages before 28 hpf, and found that arterial-venous defects in the *hNRASD12*-expressing embryos appeared as early as 20 hpf (Figure 7A-L), when the identity of arterial-venous cell fate is just established [25], indicating N-Ras signaling regulates the specification of arterial and venous cells before the arterial-venous segregation and onset of circulation. In addition, previous studies have revealed an important role of the angioblasts migration between 14 and 18 hpf in the arterial-venous specification [23,32]. To examine whether the constitutively activated N-Ras signaling affects the medial migration of angioblasts, we used *flk1* as a probe to trace this process. The result showed the medial migration began at 14–15 hpf, and nearly all angioblasts arrived at the midline at 17–18 hpf in the control embryo (Figure 8A-E), and *hNRASD12*-expressing embryo showed a similar pattern (Figure 8F-J), indicating the activated N-Ras signaling specifically affects the arterial-venous specification in an angioblasts-medial-migration-independent manner.

To further confirm that the defects in the assembly of the blood vessels and the arterial and venous fate decisions are specifically induced by the transgenic expression of h*NRASD12*, we knocked down the *hNRASD12* expression using hNRAS MO in the h*NRASD12* embryos. The trunk vasculature was imaged under a confocal microscopy and arterial and venous cells in the region were evaluated by WISH analysis of arterial-venous cells specific markers. Compared to the control MO, the hNRAS MO efficiently rescued both the defective assembly of blood vessels (Additional file 5: Figure S4A) and the unbalanced arterial-venous fates of the endothelial cells (Additional file 5: Figure S4B) in the trunk region of *hNRASD12* embryo, indicating the defective vasculogenesis in the transgenic embryos is specifically caused by *hNRASD12* expression.

Thus we conclude that the activated N-Ras signaling by expressing *hNRASD12* in *lmo2*^+^ cells disrupts the balance of the arterial-venous specification, where the venous fate decision is enhanced while the arterial specification is repressed, which in turn causes abnormal assembly of main blood vessels and the defect in the development of intersegmental vessels, leading to blockage of blood circulation and embryonic lethality. Nevertheless, expression of *hNRASD12* affects neither the endothelial specification from mesoderm cells, the medial migration of angioblasts, nor the proliferation of endothelial cells.

The hierarchical signaling molecules, including sonic hedgehog (Shh), vascular endothelial growth factor A (VEGFA), phospholipase C gamma-1 (Plcg1), and notch have been shown to be essential for the arterial cell fate decision [33]; while chicken ovalbumin upstream promoter-transcription factor II (COUP-TFII), also known as Nuclear Receptor 2F2 or NR2F2, is indispensable for the venous specification [33]. To figure out whether *hNRASD12* regulates the transcriptional expression of those genes, we performed WISH analysis with riboprobes of these arterial-venous regulatory genes, and found no significant difference between the control and *hNRASD12* embryos (Additional file 5: Figure S5A-F and S5A’-F’).

Previous studies have shown that forced expression of *vegfaa*_*121*_ in wild-type embryo could transform all the endothelial cells into *ephrinB2*^*+*^ arterial cells [34], however, we observed the failure of *hNRASD12* embryos to respond to exogenous expression of *vegfaa121* (Additional file 5: Figure S6A-E), indicating that N-Ras signaling functions downstream of vegfa to negatively regulate the vegfa induced arterial development.

Considering notch signaling has been proved to act downstream of *shh-vegfa* signaling axis to regulate the arterial endothelial differentiation in zebrafish [35], we tried to rescue the *hNRASD12*-induced defective arteriogenesis by injecting a transient transgenic construct into the *hNRASD12* embryos, in which a constitutively activated form of notch1 containing only the intracellular domain (referred as NICD1) was driven by the zebrafish *flk1* promoter, however, no recovery of arteriogenesis was observed in those injected embryos (data not shown), implying defects caused by activated Ras is not through disrupting the notch signaling.

### Functional conservation of human and zebrafish N-Ras signaling in vasculogenesis

To assess the conservation between the human and zebrafish N-Ras signaling, we compared the protein sequences of the human, mouse and zebrafish N-RAS, and found that there is a high degree of sequence similarity between the zebrafish and mammalian N-RAS with a high identity of 95% in the critical H_N_K_Ras_like domain (G domain) (Additional file 5: Figure S7). To further determine whether the activated zebrafish N-Ras signaling has a conserved function in the vasculogenesis as human N-Ras, we generated both *lmo2:EGFP-2A-hNRASD12* and *lmo2:EGFP-2A-zNRASD12* transgenic constructs, in which the zebrafish *lmo2* promoter was used to transiently direct the specific expression of activating mutant of human and zebrafish N-Ras in blood and vasculature respectively. The WISH analysis showed that both the *hNRASD12* and *zNRASD12* transcripts were appropriately expressed in the ICM region (Additional file 5: Figure S8A), similar to the expression pattern of endogenous *lmo2* gene (Figure 1D), and the expression of the human and zebrafish NRASD12 proteins was also confirmed via western blotting assay (Additional file 5: Figure S8B). The trunk vasculature of embryos expressing the *hNRASD12* or *zNRASD12* was characterized under a confocal microscopy. And compared to the embryos injected with *lmo2:EGFP-2A*, both the *hNRASD12* and *zNRASD12*-expressing embryos had only one vascular tube in the trunk (Additional file 5: Figure S8C). Additional WISH analysis of the arterial marker *ephrinB2* and venous marker *flt4* genes showed large expansion of venous cells population and nearly lack of the arterial cells in the trunk region (Additional file 5: Figure S8C). These results strongly suggest a conserved function between activated human and zebrafish N-RAS and that the N-Ras signaling participate in regulating the zebrafish vascular development.

## Discussion

In this study, we showed that transgenic overexpression of activated human *NRAS* (*hNRASD12*) in zebrafish *lmo2*^+^ cells caused un-circulated vasculature, but had no impact on the primitive hematopoiesis during embryogenesis. In particular, the expression of *hNRASD12* promoted the venous fate at the expense of arterial fate during arterial-venous specification, indicating an important novel role of activated Ras signaling in vascular morphogenesis.

Our whole mount *in situ* hybridization (WISH) and quantitative RT-PCR analysis of the expression of the hematopoietic markers showed the expression of *hNRASD12* in the *lmo2*^+^ hemangioblasts neither affected the specification or differentiation of the primitive hematopoietic cells, nor induced any leukemic phenotypes. However, the mice models expressing *hNRASD12* exhibited leukocytosis and progressed to myeloid leukemias [4-7]. The discrepancy between our zebrafish model and those mice models is likely due to the difference in the targeting cell type and the developmental window of the *NRASD12* expression. In the mice models, the expression of the *NRASD12* was induced in the adult bone marrow cells, the adult hematopoietic organs, and caused dysplastic myelopoiesis/leukemias with long latencies (> 4 weeks), which highlight the possible requirement of a definitive hematopoietic environment and the length of time to accumulate cooperative genetic mutations for the development of *NRASD12*-associated leukemia [36]. In our study, the expression of *hNRASD12* was activated during the transient primitive hematopoietic stage and embryos failed to develop through the definitive hematopoietic stages due to lack of definitive hematopoiesis as a result of the defective vasculogenesis, and early embryonic lethality (5–8 days post fertilization), preventing the occurrence of the myeloid disorders and leukemias. Our results supported the concept that leukemogenesis is a multistep process involving cooperating genetic mutations [37], the elevation of RAS signaling by the expression of *hNRASD12* alone in the short-lived primitive hematopoietic cells was insufficient for leukemogenesis.

Whether the hyperactive Ras signaling caused by inactivating RASA1 mutations in Capillary Malformation-Arteriovenous Malformation (CM-AVM) functions through an endothelial cell-autonomous manner is still unknown [9]. Here we showed that the expression of oncogenic human N-Ras in the *lmo2*^+^ cells disrupted arterial-venous specification and angiogenesis in zebrafish embryos, indicating an endothelial cell-autonomous role of oncogenic ras signaling in vasculogenesis. Consistently, the *rasa1*-deficient mice embryos are embryonic lethal and exhibit atrophic dorsal aorta and disorganized patterns of the intersegmental arteries [38]. The EphrinB2/EphB4 signaling [10,11] and notch pathway [12] are involved in the pathogenesis of Arteriovenous Malformation (AVM), suggesting the direct genetic connection between the arterial-venous specification and CM-AVM/AVM. In our study, the transgenic expression of *hNRASD12* induces defective arterial-venous specification in zebrafish, and this vertebrate genetic model may help us to further dissect the pathogenesis of CM-AVM or AVM.

In mice, it has been demonstrated that the germ line oncogenic Ras expression leads to embryonic lethality [6,39], where the *Protamine-Cre* (PrmCre) mice mediates constitutive and ubiquitous expression of oncogenic *KRASD12* (floxed knock-in) [39], and *mox2-Cre* mice mediates germ line expression of endogenous oncogenic *NRASD12*[6], however, the cellular and pathological mechanisms for the embryonic lethality in those studies are unknown. Here we show that the expression of oncogenic *hNRASD12* during zebrafish embryogenesis induces embryonic lethality through disrupting the arterial-venous cell fate decision, offering a possible mechanistic explanation for the oncogenic Ras signaling induced embryonic lethality. Nevertheless, the phenotypes of endothelial development need to be further examined in those mice models [6,39].

The zebrafish has emerged as the first vertebrate model organism that is suitable for large-scaled whole-animal small chemical molecule screening, contributing to several aspects of the drug development process, including target identification, disease modeling, lead discovery and toxicology [40]. The oncogenic *NRASD12*-expressing mice develop leukemias in adult stage [4-7], and are not suitable for drug screening due to the cost and the large size of an individual, while the *NRASD12* transgenic zebrafish embryos may be helpful for us to perform large-scaled whole-animal small chemical molecule screening to find oncogenic *NRASD12*-associated modulators using a developmental stage-controlled expression of oncogenic NRASD12.

## Conclusions

In conclusion, we report firstly the constitutively activated N-Ras signaling regulates the arterial-venous specification in vertebrate, and also established an oncogenic *hNRASD12* induced animal disease model that is feasible for high-throughput whole-animal drug screening and validating the functionality of *NRASD12* modulators as potential therapeutics for a variety of human diseases caused by oncogenic mutant RAS.

## Methods

### Fish care

Zebrafish maintenance, breeding, and staging were performed as previously described [41]. The zebrafish experiments were approved by the Animal Experimentation Committee of Shanghai Institutes for Biological Sciences and institutional review board of Institute of Health Sciences.

### Generation of *Tg(β-actin:LDL-hNRASD12)* transgenic line

*Human NRASD12* (*hNRASD12*) was obtained by PCR from a myc-tagged *NRASD12* in a retroviral vector [4] and cloned into pCS2+ vector at the ClaI and XhoI sites to get the *hNRASD12-pCS2+* construct. Then the *hNRASD12-SV40* fragment was excised from *hNRASD12-pCS2+* plasmid by ClaI and KpnI and cloned into the same sites of *β-actin-loxP-DsRed-SV40-loxP-PBSK-I-SceI* plasmid [16] to get the *β-actin-loxP-DsRed-SV40-loxP-hNRASD12*-*PBSK-I-SceI* transgenic construct. Then this construct was co-injected with I-SceI meganuclease (New England Biolabs) into the fertilized zebrafish eggs at one cell stage. The injected embryos were raised to adulthood and crossed to wild-type fish to generate the F1 progeny, and the transgenic founders were identified by screening for the F1 embryos, expressing DsRed fluorescence at 24 hours post fertilization (hpf). We raised the DsRed^+^ F1 embryos to adulthood to establish the stable transgenic lines.

### Transient transgenic constructs

The zebrafish *lmo2* promoter of 2.5 kbp was obtained by PCR amplification from the *lmo2-Cre-PBSK-I-SceI* plasmid [16] and cloned into the *EGFP-2A-pDestTol2* plasmid (Z.W.Dong et al., unpublished data) at the XhoI and BamHI sites to get the *lmo2-EGFP-2A-pDestTol2* construct. Then the human *NRASD12* (*hNRASD12*) and zebrafish *NRASD12* (*zNRASD12*) fragments were obtained by PCR from the *β-actin-loxP-DsRed-SV40-loxP-hNRASD12*-*PBSK-I-SceI* plasmid and cDNA from the wild-type embryos respectively, and cloned into the *lmo2-EGFP-2A-pDestTol2* construct at the XmaI and SalI sites to get the *lmo2-EGFP-2A-hNRASD12/ zNRASD12-pDestTol2* constructs. Of note, the forward primer for the amplification of zebrafish *NRASD12* contains mutations at the codon 12 from GGA to GAT, resulting in the substitution of glycine (G) to aspartic acid (D). Then the *lmo2-EGFP-2A-pDestTol2*, *lmo2-EGFP-2A-hNRASD12-pDestTol2* and *lmo2-EGFP-2A-zNRASD12- pDestTol2* transgenic plasmids (25 ng/ul) were individually co-injected with KCl (0.2 M) and Tol2 transposase mRNA (25 ng/ul) as previously described [42] into the *Tg(flk1:mCherry)* or wild-type zebrafish embryos at one cell stage.

To generate the construct of the zebrafish *flk1* promoter driven *NICD1*, the HA-tagged *NICD1* fragment was obtained by PCR from the PME-*NICD1* (zebrafish) plasmid and cloned into the *flk1-PolyA-pBSKI2* plasmid at the BamHI and EcoRI sites to get the *flk1-HA-NICD1-PolyA-pBSKI2* construct. Then the construct was co-injected with I-SceI meganuclease into the *hNRASD12* transgenic embryos at one cell stage.

### Morpholino (MO)

Embryos were injected with 2 nl human NRAS ATG MO (Gene Tools): 5’-ACCAGTTTGTACTCAGTCATATCGA-3’. The efficiency of hNRAS MO was confirmed both by western blotting and co-injection with *hNRAS*^*1-60*^*-GFP* mRNA reporter containing the targeting sites of hNRAS MO.

### Preparation of mRNAs and antisense probes, whole-mount *in situ* hybridization (WISH)

A 60 bp fragment of human *NRAS* that contains the hNRAS ATG MO binding sites was fused to the N terminal of GFP in the pCS2+ vector to get the *hNRAS*^*1-60*^*-GFP-pCS2+* construct. The *hNRAS*^*1-60*^*-GFP*, zebrafish *vegfaa*_*121*_[35] and Tol2 transposase [43] mRNAs were synthesized with mMESSAGE mMACHINE Kit (Ambion).

The digoxigenin- or fluorescein-labelled antisense probes used for WISH analysis in this study includes *lmo2*[16], (*shh*, *notch1*) [17], *vegfaa*_*121*_[35], (*plcg1*, *pu.1*, *l-plastin*, *mpo*, *gata1*, *αe1 globin*, *flk1*) [44], (*cmyb*, *runx1*) [45], (*ephrinB2*, *hRT*, *grl*, *notch3*) [23], (*dab2*, *ephB4 and flt4*) [28]*.* And the *hNRASD12*, *zNRASD12*, *dll4* and *nr2f2* were cloned into pCS2+ plasmid and their digoxigenin-labelled antisense probes were synthesized with T3 polymerase (Ambion).

Single- and 2-color WISH were performed as described previously [17]. Embryos were mounted in 3% methylcellulose and captured under the Nikon SMZ1500 microscope equipped with a Nikon DXM1200F digital camera and ACT-1 software.

### Genomic DNA isolation and genotyping

Embryos were incubated in lysis buffer (Tris–HCl 1 M, pH 8.3; KCl 1 M; Tween 20 10%; NP40 10%) and protease K (10 mg/ml) at 55°C overnight. Then 3700 rpm for 10 min at 4°C, and the supernatant was subject to genomic PCR with specific primers for *Cre* and *hNRASD12*.

### Real-time Quantitative PCR

Total RNAs were extracted from 20 zebrafish embryos using Trizol reagent (Invitrogen). RNA was reverse-transcribed using random hexamers and SuperScript III Reverse Transcriptase (Invitrogen). 2×PCR Mix (TaKaRa, Premix Ex Taq™) containing SYBR Green I was used for the real-time quantitative PCR analysis with the Applied Biosystems 7900HT Fast Real-Time PCR System. The relative expression values were normalized against the internal control *gapdh* (qPCR primer sequences were listed in Additional file 5: Table S1).

### Western blotting and immunofluorescence

The western blotting assay was performed as described previously [17] with antibodies: human NRAS (Santa Cruz), GAPDH (Cell Signaling Technology). Immunohistochemistry was performed as described previously [25], and the trunk of embryo was transversely sectioned with a Leica VT1000S vibratome into 50–100 μm sections, and F-actin was stained with Phalloidin-TRITC (0.007 μM, Sigma), and GFP antibody (Invitrogen, A6455) was used. Sections were mounted with Dako-fluorescent mounting media (Dako North America) prior to imaging with an Olympus FV 1000 confocal microscopy.

### Fluorescence microangiography

The fluorescein-coupled latex beads (Molecular Probes) were injected into the inflow tract of the atrium of the zebrafish embryo at 30 hpf with a dose of 2 nl. The beads flowed with blood circulation and labeled all the vascular vessels of wild-type embryos in 5 minutes.

### Flow cytometry analysis

Embryos at 28 hpf were dissected in DPBS (Invitrogen) after dechorionation and deyolking [46], and digested with 1× trypsin/EDTA (Life Technologies) for 30 min at 28.5°C. Single cell suspension was obtained by centrifugation at 400 g for 5 min, washed twice with 0.9XPBS/5%FBS, and passed through a 40 μm nylon mesh filter. Fluorescence-activated cell sorting was performed with FACSAria (BD Biosciences) to quantify the frequency of the GFP^+^ endothelial cells.

### Image analysis

Embryos were imaged under an Olympus FV 1000 confocal microscopy equipped with the FV10-ASW version3 software.

### Statistical analysis

The values were represented as mean ± SEM. Two-tailed students’t-tests were used for two groups comparison analysis, and P < 0.05 was considered to be significant. The bar charts were used to reflect the alterations of experimental data.

## Competing interests

The authors declare that they have no competing interests.

## Authors’ contributions

CGR performed experiments and analyzed data. LW, XEJ, YJL, ZWD, YZ(Yong Zhou), YJ, YC, and MD assisted with experiments. TXL, WJP, YZ(Yi Zhou), RBR, and CGR designed the research plan and wrote the paper. All authors were involved in the writing and final approval of the manuscript.

## Supplementary Material

Additional file 1 **Movie S3.** The blood circulation in the trunk of β-actin:LDL-hNRASD12;lmo2:Cre embryo at 28 hpf. Click here for file

Additional file 2 **Movie S4.** The blood circulation in the head of β-actin:LDL-hNRASD12;lmo2:Cre embryo at 28 hpf. Click here for file

Additional file 3 **Movie S1.** The blood circulation in the trunk of β-actin:LDL-hNRASD12;wild-type embryo at 28 hpf. Click here for file

Additional file 4 **Movie S2.** The blood circulation in the head of β-actin:LDL-hNRASD12;wild-type embryo at 28 hpf. Click here for file

Additional file 5 Supplementary figures and table. Click here for file
