# Supplementary material for: Activated N-Ras signaling regulates arterial-venous specification in zebrafish
Source: J Hematol Oncol. 2013 May 12;6:34. doi: 10.1186/1756-8722-6-34 (PMC3658992; doi:10.1186/1756-8722-6-34)
Supplement: Additional file 5 — Supplementary figures and table. [file 1756-8722-6-34-S5.doc]

**Additional file 1. Supplementary figures and table.**

**Activated N-Ras signaling regulates arterial-venous specification in zebrafish**

Chun-Guang Ren, Lei Wang, Xiao-E Jia, Yi-Jie Liu, Zhi-Wei Dong, Yi Jin, Yi Chen, Min Deng, Yi Zhou, Rui-Bao Ren, Wei-Jun Pan, Ting-Xi Liu

**Supplementary Figures and Table**

**Figure S1. Efficiency validation of hNRAS morpholino (MO).**

**Figure S2. The defective cardiovascular system in the *hNRASD12*-expressing embryos.**

**Figure S3. *Lmo2* promoter driven *hNRASD12* expression suppresses arterial identity in vasculogenesis.**

**Figure S4. The defective vasculogenesis is specifically caused by *hNRASD12* expression.**

**Figure S5. The expression patterns of arterial-venous regulators in control and *hNRASD12*-expressing embryos.**

**Figure S6. Activated N-Ras signaling functions downstream of the VEGFA signaling to negatively regulate the exogenous vegfa induced arteriogenesis.**

**Figure S7.** **Alignment of amino acid sequences of human, mouse, and zebrafish N-RAS proteins.**

**Figure S8. Functional conservation of human and zebrafish N-Ras signaling in vasculogenesis.**

**Table S1. Primer list for cloning, genomic PCR and qPCR.**

**
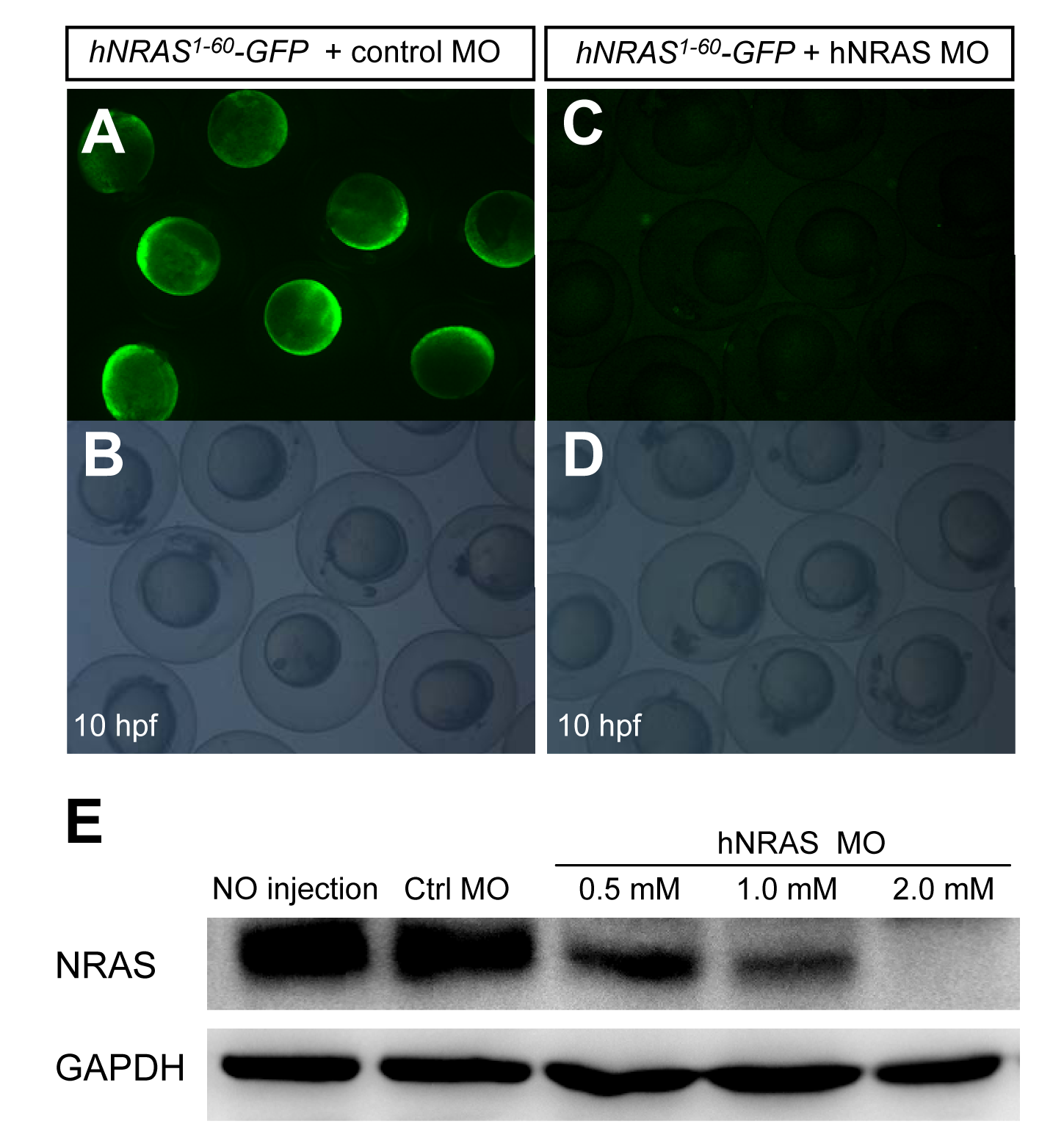
**

**Figure S1. Efficiency validation of hNRAS morpholino (MO).** (A-D) Fluorescent and bright images of zebrafish embryos injected with *hNRAS^1-60^-GFP* reporter mRNA and control (A, B)/hNRAS MO (C, D). (E) Dose-dependent inhibition of hNRAS protein expression by hNRAS MO in the *hNRASD12* embryos at 28 hpf.

**
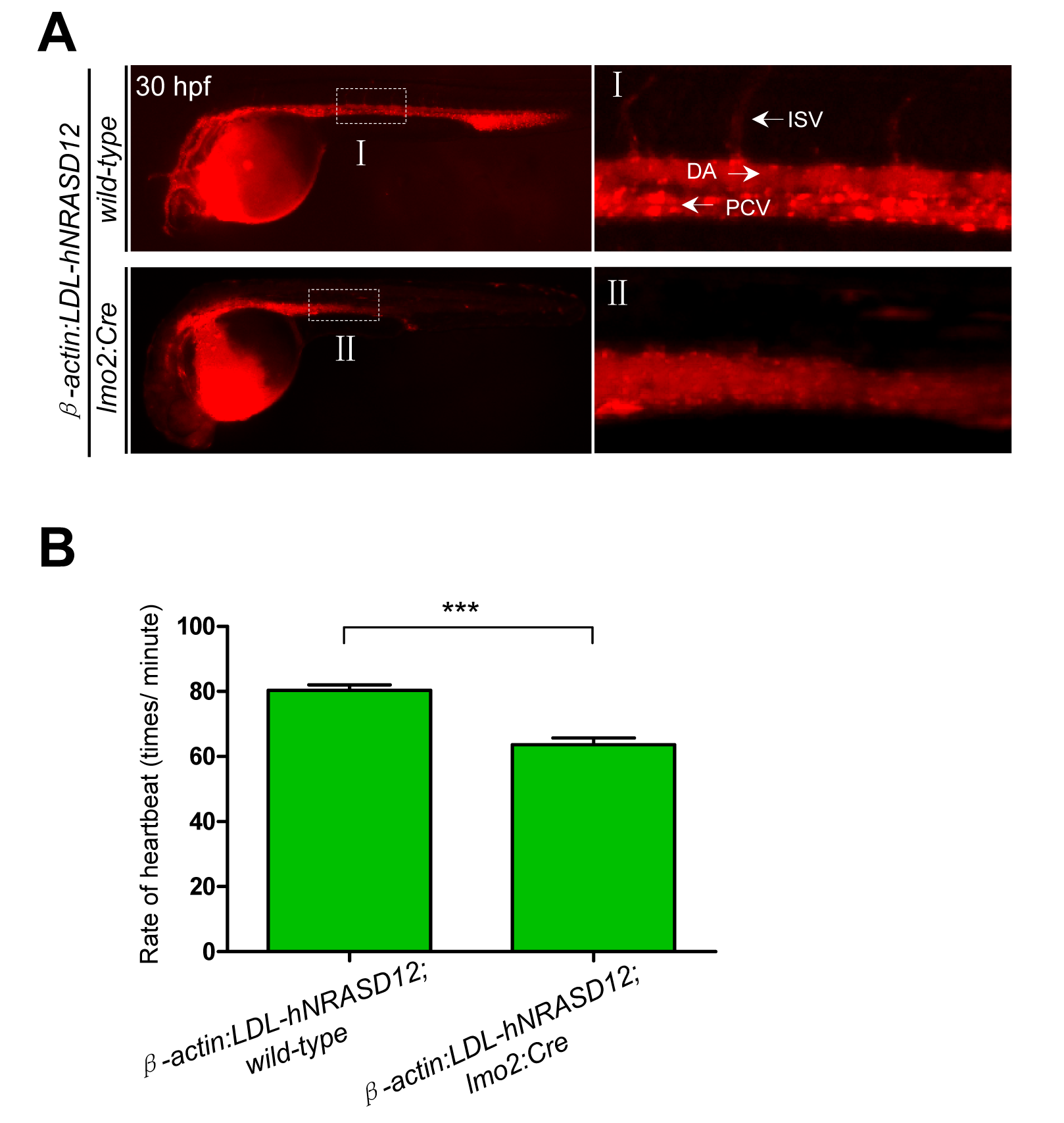
**

**Figure S2. The defective cardiovascular system in the *hNRASD12*-expressing embryos.** (A) The fluorescence microangiography of the cardiovascular system in the control (upper panel) and *hNRASD12* embryos (lower panel) at 30 hpf. DA, dorsal aorta; PCV，posterior cardinal vein; ISV, intersegmental vessel. (B) The rates of heartbeats (times/min) in the control and *hNRASD12* embryos at 30 hpf. The asterisks denote the significant difference of heartbeats between the control and *hNRASD12* embryos (p< 0.001).

**
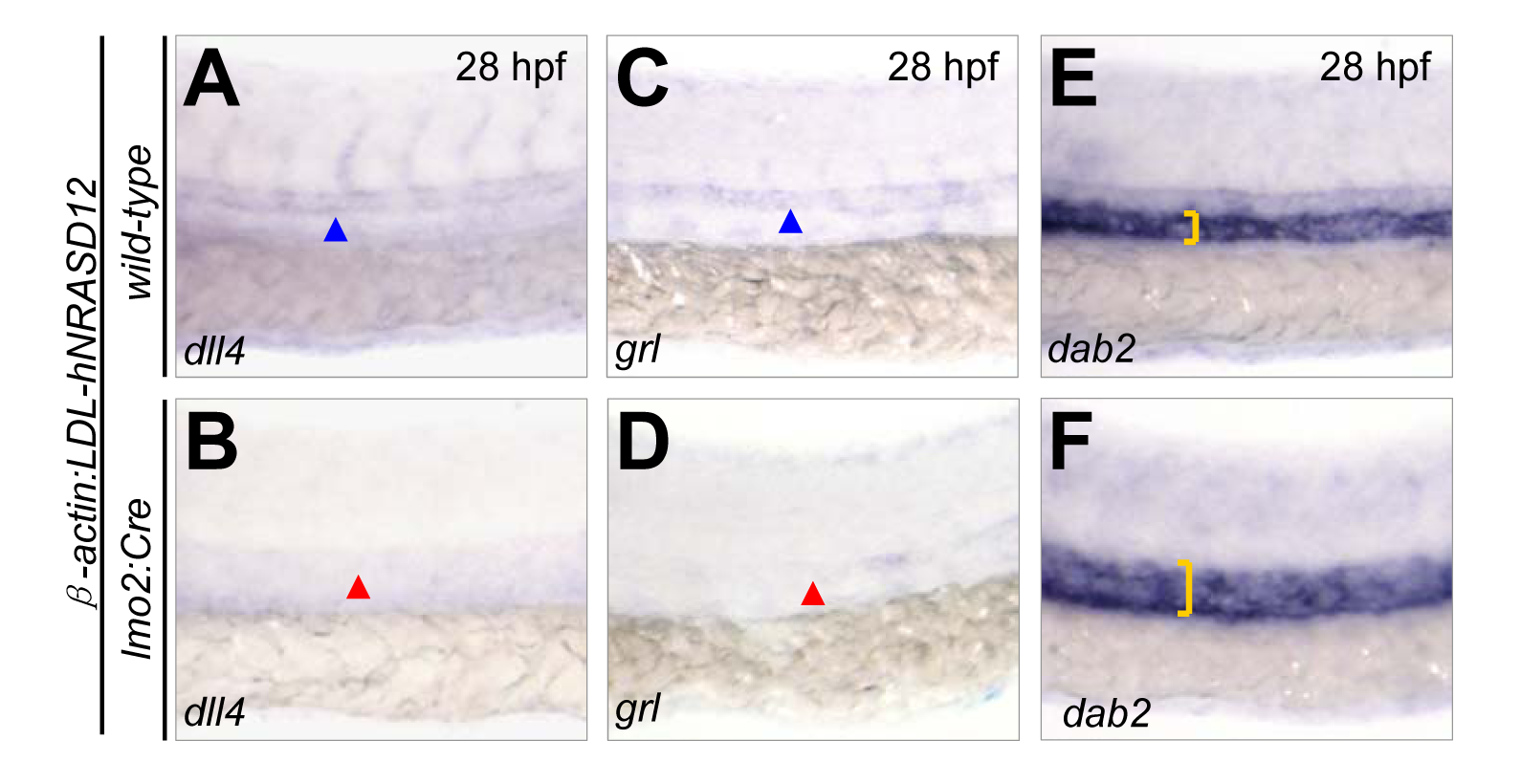
**

**Figure S3. *Lmo2* promoter driven *hNRASD12* expression suppresses arterial identity in vasculogenesis.** (A-D) WISH analysis of *dll4^+^* and *grl^+^* arterial cells in the trunk of control (A, C, blue triangles) and *hNRASD12* embryos (B, D, red triangles) at 28 hpf. (E-F) WISH analysis of *dab2^+^* venous cells in the trunk of control (E) and *hNRASD12* embryos (F) at 28 hpf. Yellow square brackets denote the width of the *dab2^+^* cellular strip.

**
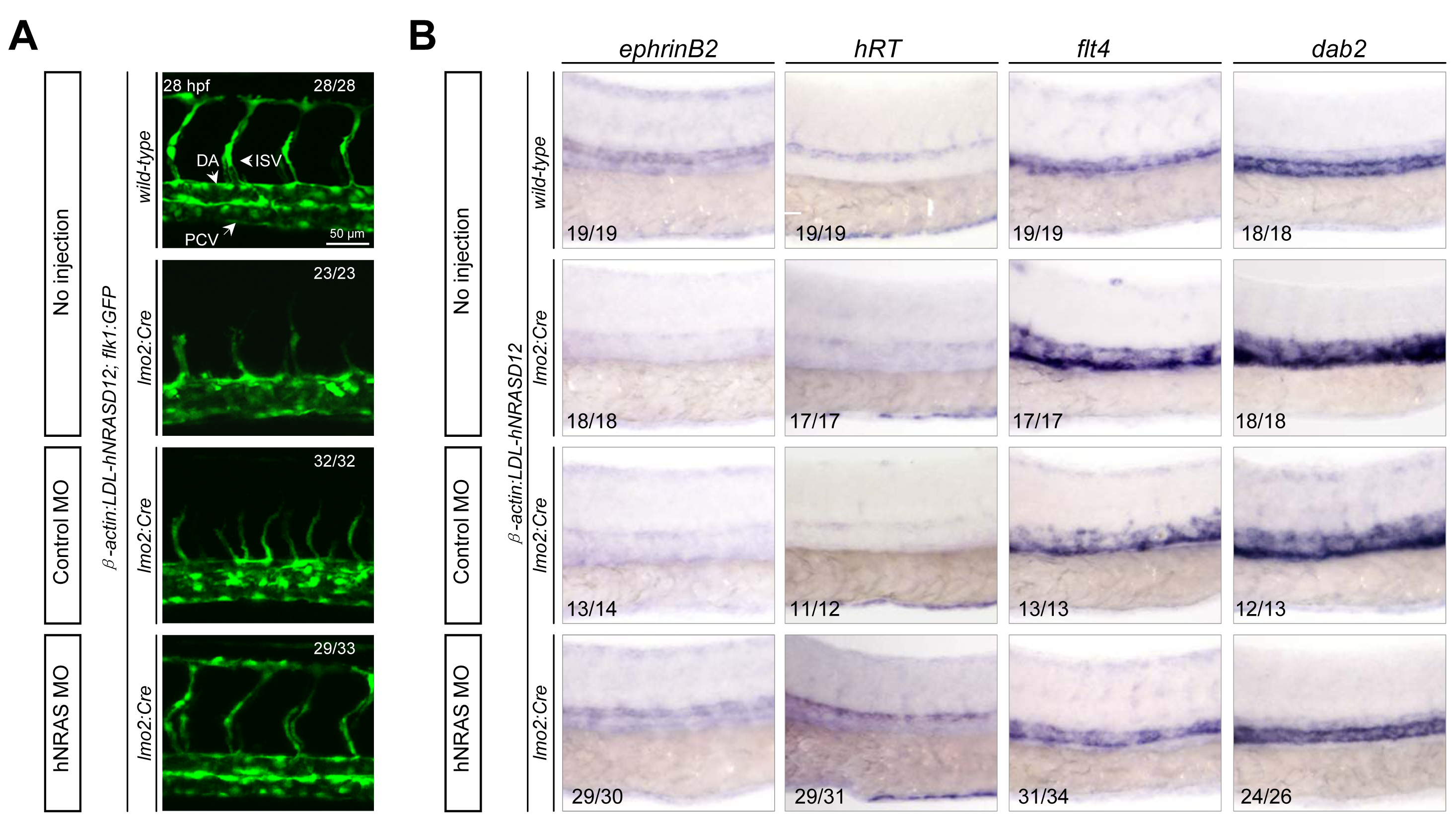
**

**Figure S4.** **The defective vasculogenesis is specifically caused by *hNRASD12* expression.** (A) Confocal images of trunk vasculature of living *control;flk1:GFP* and *hNRASD12;flk1:GFP* embryos at 28 hpf injected with control/hNRAS MO. DA, dorsal aorta; PCV, posterior cardinal vein; ISV, intersegmental vessel. (B) WISH analysis of the arterial (*ephrinB2^+^*, *hRT^+^*) and venous (*flt4^+^*, *dab2^+^*) cells in the trunk of control and *hNRASD12* embryos at 28 hpf injected with control/hNRAS MO.

**
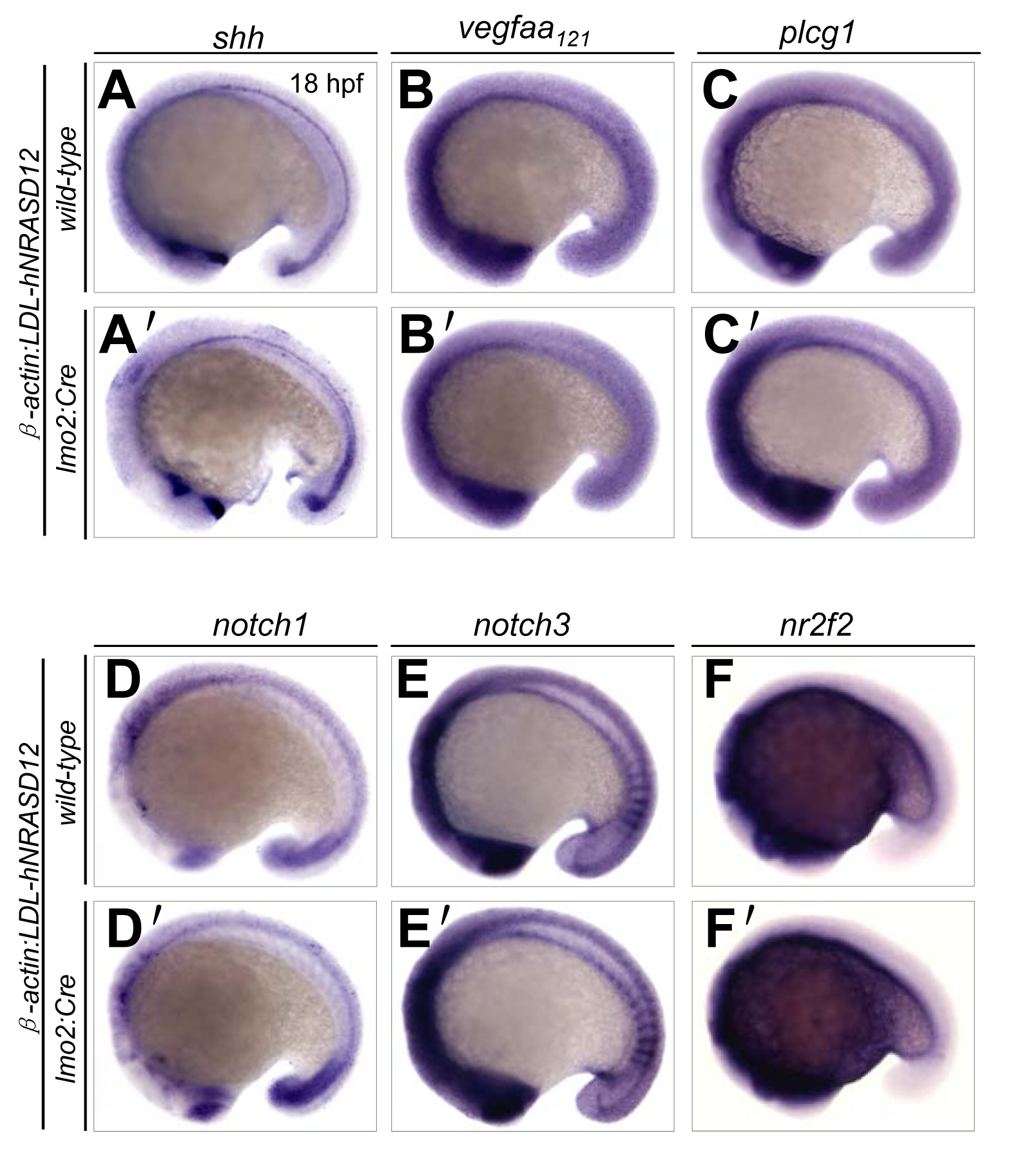
**

**Figure S5. The expression patterns of arterial-venous regulators in control and *hNRASD12*-expressing embryos.** (A-F′) The expression patterns of *sonic hedgehog* (*shh*), *vascular endothelial growth factor aa 121* (*vegfaa_121_*), *phospholipase C gamma-1* (*plcg1*), *notch1*, *notch3*, and *nr2f2* transcripts in control (A-F) and *hNRASD12* embryos (A′-F′) at 18 hpf were analyzed by WISH assay.

**
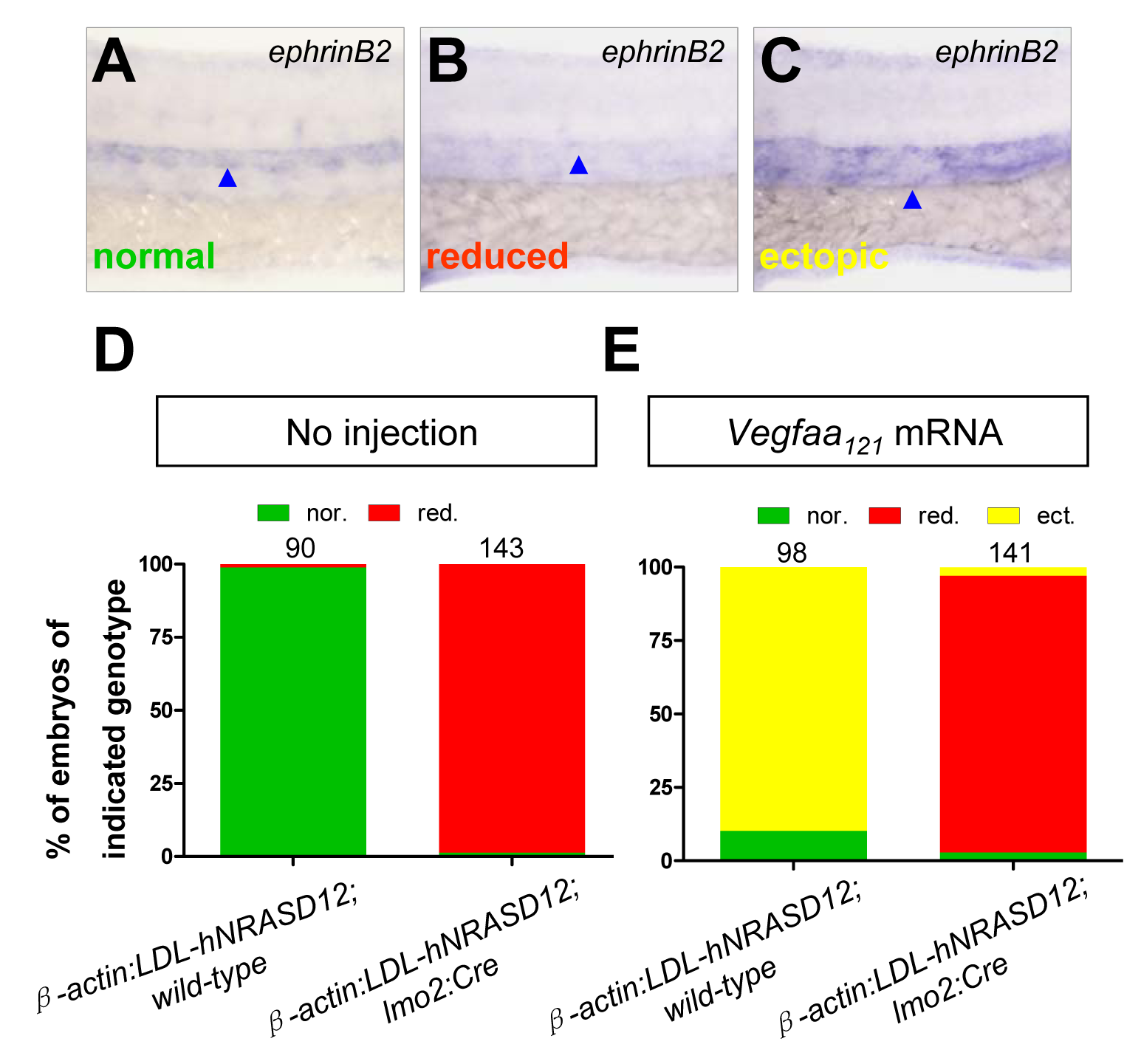
**

**Figure S6. Activated N-Ras signaling functions downstream of the VEGFA signaling to negatively regulate the exogenous vegfa induced arteriogenesis.** (A-C) The representative patterns of the normal (A), reduced (B) and ectopic (C) *ephrinB2^+^* arterial cells. (D) Proportion of uninjected embryos displaying *ephrinB2* expression. (E) Proportion of genotyped embryos injected with *vegfaa_121_* mRNA that display normal, ectopic, or reduced *ephrinB2* expression within the trunk blood vessels. The numbers on the top of the bars indicate the amount of the embryos being counted, and representative results obtained from three independent experiments.


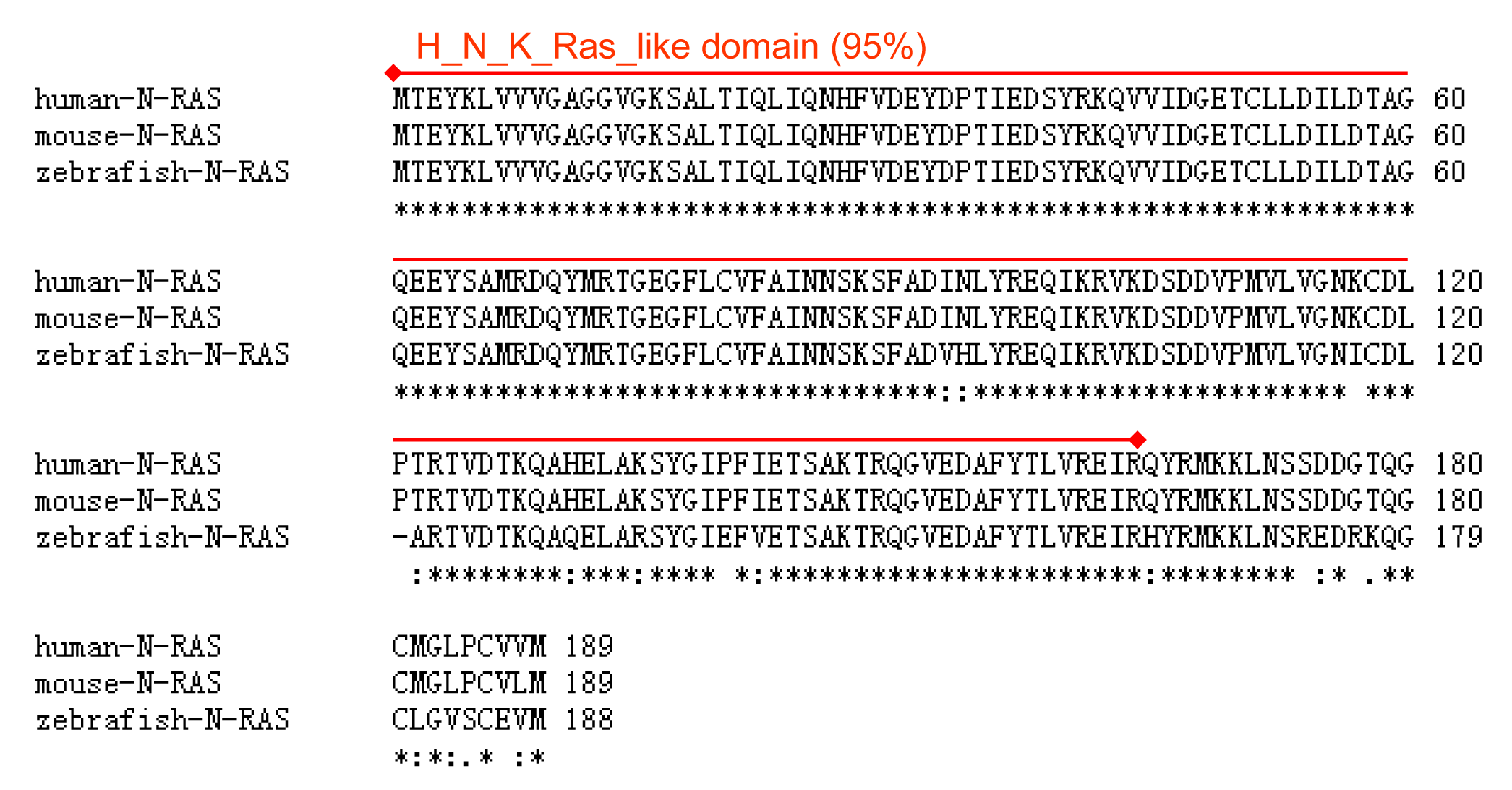


**Figure S7.** **Amino acid sequences alignment of human, mouse, and zebrafish N-RAS proteins.** Sequence alignment was performed with ClustalW2 program. The percentages in the brackets denote the amino acid identities of the H_N_K_Ras_like domain (G domain) highlighted with red line.


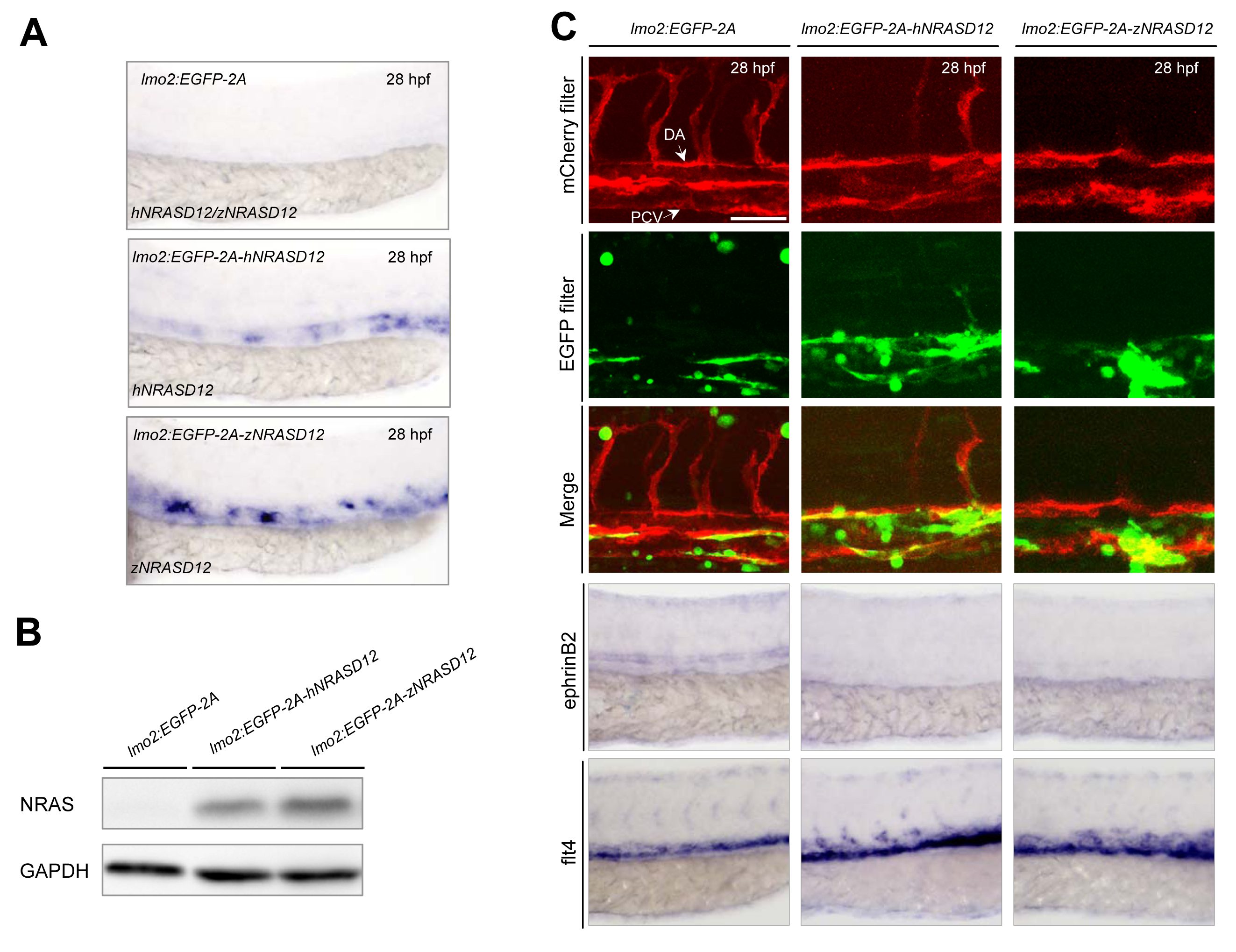


**Figure S8.** **Functional conservation of human and zebrafish N-Ras signaling in vasculogenesis.** (A) WISH analysis of the expression of the human and zebrafish *NRASD12* transcripts in the wild-type embryos (28hpf) injected with *lmo2:EGFP-2A*, *lmo2:EGFP-2A-hNRASD12*, and *lmo2:EGFP-2A-zNRASD12* transient transgenic constructs. (B) Immunoblotting analysis on the expression of NRAS protein in the *lmo2:EGFP-2A-hNRASD12*, and *lmo2:EGFP-2A-zNRASD12* transient injected embryos at 28 hpf. (C) Confocal images of trunk vasculature of *Tg(flk1:mCherry)* embryos at 28 hpf injected with the above three transgenic constructs, and WISH analysis of the arterial (*ephrinB2^+^*) and venous (*flt4^+^*) cells in the wild-type embryos injected with the three constructs. DA, dorsal aorta; PCV, posterior cardinal vein. Scale bars, 60 μm.

| **cloning** | **Forward 5'→3'** | **Reverse 5'→3'** |
| --- | --- | --- |
| human NRASD12 (driven by βactin promoter) | CACGAATTCACTGAGTACAAACTGGTGG | CTGCTCGAGTTACATCACCACACATGGC |
| human NRASD12 (driven by lmo2 promoter) | ATTACCCGGGGCCACCATGACTGAGTACAAACTGGT | GTCAGTCGACTTACATCACCACACATGGCA |
| zebrafish lmo2 promoter | GCCGCTCGAGGAATTCTTTATACGATGGAA | GTCAGGATCCTCTCTGTGTCCCGTTATTGT |
| zebrafish NRASD12 | ATTACCCGGGGCCACCATGACTGAGTATAAGCTGGTTGTTGTGGGAGCAGATGGTGTTGGGAAGAGCGCGTTAA | ATTCGTCGACTCACATGACTTCACAGGACACGC |
| dll4 | GCGTGAATTCTGCAGGACTTTCTTCCGAGT | AATACTCGAGACCCCAGCCCTCTTTACAGT |
| nr2f2 | CCTCGAATTCATGGCAATGGTAGTGTGGAG | AAGTCTCGAGCTAGTAGGAATGGCAGTGCAGTG |
| HA-NICD1 | GCTCGGATCCATGTACCCATACGATGTTCCAGATTACGCTAACGAACCCAAAAAGAAGAGGAGAGAACCA | GCTCGAATTCCTACTTGAAGGCTTCTGGAA |
|  |  |  |
| **genomic PCR** | **Forward 5'→3'** | **Reverse 5'→3'** |
| cre | AAAATTTGCCTGCATTACCG | TTGCCCCTGTTTCACTATCC |
| human NRASD12 | CGCACTGACAATCCAGCTAA | TCGCTTAATCTGCTCCCTGT |
|  |  |  |
| **qPCR** | **Forward 5'→3'** | **Reverse 5'→3'** |
| lmo2 | AAATGAGGAGCCGGTGGAT | GCTCGATGGCCTTCAGAAA |
| gata1 | TCTGAGCCTTCTCGTTGGG | CTCTGGACGCTGGTGGAATA |
| αe1 globin | CCAGGATGTTGATTGTCTAC | CAGTCTTGCCGTGTTTC |
| pu.1 | AGGAGTGTATGAGAGACCACATCAG | ATTTCGCAGAAGGTCAAGCA |
| l-plastin | TGTCTGTGCCCGACACCAT | GGCGGAGGCAGAGTTCAG |
| mpo | TCAATATGAGGACGCCGTTTCT | GAATGCGATTGGAAACCAGTCT |
| gapdh | CCAACTGCCTGGCTCCTT | CCCATCAACGGTCTTCTGTG |

**Table S1.** **Primer list for cloning, genomic PCR and qPCR.**
